# Social Integrating Robots Suggest Mitigation Strategies for Ecosystem Decay

**DOI:** 10.3389/fbioe.2021.612605

**Published:** 2021-05-24

**Authors:** Thomas Schmickl, Martina Szopek, Francesco Mondada, Rob Mills, Martin Stefanec, Daniel N. Hofstadler, Dajana Lazic, Rafael Barmak, Frank Bonnet, Payam Zahadat

**Affiliations:** ^1^Artificial Life Laboratory of the Institute of Biology, University of Graz, Graz, Austria; ^2^Mobile Robotic Systems Group, School of Engineering and School of Computer and Communication Sciences, École Polytechnique Fédérale de Lausanne, Lausanne, Switzerland; ^3^BioISI, Faculdade de Ciências da Universidade de Lisboa, Lisbon, Portugal; ^4^Department of Computer Science, IT University of Copenhagen, Copenhagen, Denmark

**Keywords:** robot–animal interaction, robot–organism interaction, biohybrid systems, biomimicry, organismic augmentation, ecosystem collapse

## Abstract

We develop here a novel hypothesis that may generate a general research framework of how autonomous robots may act as a future contingency to counteract the ongoing ecological mass extinction process. We showcase several research projects that have undertaken first steps to generate the required prerequisites for such a technology-based conservation biology approach. Our main idea is to stabilise and support broken ecosystems by introducing artificial members, robots, that are able to blend into the ecosystem’s regulatory feedback loops and can modulate natural organisms’ local densities through participation in those feedback loops. These robots are able to inject information that can be gathered using technology and to help the system in processing available information with technology. In order to understand the key principles of how these robots are capable of modulating the behaviour of large populations of living organisms based on interacting with just a few individuals, we develop novel mathematical models that focus on important behavioural feedback loops. These loops produce relevant group-level effects, allowing for robotic modulation of collective decision making in social organisms. A general understanding of such systems through mathematical models is necessary for designing future organism-interacting robots in an informed and structured way, which maximises the desired output from a minimum of intervention. Such models also help to unveil the commonalities and specificities of the individual implementations and allow predicting the outcomes of microscopic behavioural mechanisms on the ultimate macroscopic-level effects. We found that very similar models of interaction can be successfully used in multiple very different organism groups and behaviour types (honeybee aggregation, fish shoaling, and plant growth). Here we also report experimental data from biohybrid systems of robots and living organisms. Our mathematical models serve as building blocks for a deep understanding of these biohybrid systems. Only if the effects of autonomous robots onto the environment can be sufficiently well predicted can such robotic systems leave the safe space of the lab and can be applied in the wild to be able to unfold their ecosystem-stabilising potential.

## Problem Statement and Motivation

Extinction has always been a ubiquitous and important part of biological evolution shaping the “tree of life” (Haeckel, 1892) in an ever-ongoing process: species may go extinct, while new ones emerge by speciation at an equal or higher rate in parallel. This continuous diversification process has occasionally been interrupted by global mass extinction events in the past, known as the “big five” (Twitchett, 2006). During these game-changing events, significantly more species went extinct than new species emerged; thus, these mass extinctions significantly pruned the tree of life, thereby creating a sort of ecological “tabula rasa” for novel, and often more innovative, life forms to emerge. The last of these “big five” events is known to many people as the extinction of the dinosaurs, when some dinosaurs were pushed into evolving into the ancestors of the modern birds, while all classical forms of dinosaurs vanished.

In recent centuries, and even more in recent decades, we have been significantly interfering with this dynamic process of organismic diversification. Human technology induces changes in the environment, leading to rapid and massive ecosystem perturbations and alterations. These effects happen at a speed at which nature sometimes has problems catching up to in a compensatory way, as adaptation processes can take comparatively long timespans. Besides classical conservation efforts and tackling the problem by global policy changes, we should also look into the question of how modern technology can support the protection and repair of damaged ecosystems, to buy nature the time it needs to adapt naturally and to restabilise. One possible contingency strategy to support natural adaptation processes can be the introduction of robotic agents into natural ecosystems. Such robotic agents could be autonomous bio-mimetic and bio-inspired robots, which interact with natural organisms and blend into these ecosystems to be able to monitor and stabilise them from within, maybe even carrying out some interventions in case they seem necessary. In this article, we will define the problem, then expand on our hypothesis and describe several approaches towards implementing such robotic systems, as well as mathematical models and first empirical validations of our hypothesis. The objective of our article is to present a general research framework of how autonomous robots interacting with ecosystems may counteract these major issues that ecosystems are suffering, and in section “Potential Ecological Effects of Robot–Organism Interactions,” we pose a specific hypothesis regarding the manner in which robotic actors could achieve such a function (in short, through interactions with organisms that result in the stabilisation of ecosystem dynamics). We provide support towards this hypothesis with specific methodological elements through the development of predictive models and empirical illustrations.

Anthropogenic and massive ecosystem perturbations are not novel developments that are restricted to the industrial age, as human activities have changed ecosystems significantly much earlier. Early examples are the massive deforestation of Europe over the last pre-industrial centuries (Kaplan et al., 2009) or the transformation of American wildlife after the arrival of European settlers (Covington et al., 1994). Other events that are noteworthy due to their rather sudden emergence and high impact on a global scale are large cities covered in smog (Shi et al., 2016), deforestation due to acid rain (McCormick, 2013) and the hole in the ozone layer, all of which have negative effects on human health, as well as on ecosystems and global climate. While all these problems have been caused by human activities and were also a side effect of human advances in technology, these problems are also partially solved by society via the means of science and technology. Scientific research helped us to define these problems, while technology and its application provided us with solutions: for example, the hole in the Antarctic ozone layer has been in the midst of a regeneration process since 2000, after switching from harmful chemicals to ozone-friendly surrogates has been enforced by the Montreal Protocol (Solomon et al., 2016), predicted to fully and permanently close by 2050 (Schrope, 2000). The significance of these actions and an informative view on the “road not taken” is given by Prather et al. (1996).

Currently, the world is facing a massive decline in animal populations, which drives even many “keystone species” towards the threat of extinction (Barnosky et al., 2011). The numbers are so severe that scientists are already calling this trend the sixth mass extinction event (Ceballos et al., 2015, 2017; McCallum, 2015). It started with reports of honeybee collapses (Ellis et al., 2010) and continued with reports of massive insect biomass losses (Hallmann et al., 2017) and was recently extended with reports about massive vertebrate losses, e.g., in birds (Ceballos et al., 2017, 2020). Other vertebrates, e.g., fish, are also in decline through water pollution, habitat change, and over-harvesting (Hutchings and Reynolds, 2004; McCauley et al., 2015). In contrast to the natural causes that triggered the “big five” mentioned in the beginning, the current sixth massive decline of species is most likely driven by anthropogenic influences. This massive decline in diversity is expected to have dramatic consequences on humanity, as ecosystems are known to become more fragile with decreasing diversity (Nilsson and Grelsson, 1995). Thus, this decline is expected to be a self-sustaining or even a self-enhancing process.

**FIGURE 1:**
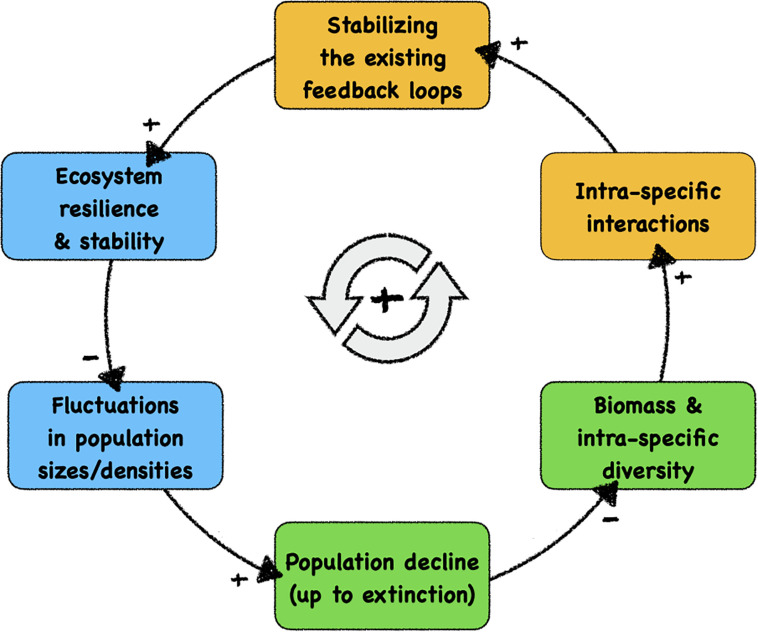
Causal loop diagram of the self-enhancing feedback loop of structural ecosystem decay, which is the likely cause of the current massive decline of biodiversity. We indicate—with background colours—the system components that can be influenced positively by autonomous technological artefacts (robots), ultimately facilitating a technology-based stabilisation of fragile ecosystems. Blue boxes: autonomous robotic probes can measure, observe, and monitor these significant properties and dynamics after being integrated into organism groups. Orange boxes: autonomous robotic agents can modulate these significant processes after being integrated into the relevant organism groups. Green boxes: natural variables in ecosystems that are targetted by our proposed contingency strategy. At the causal link arrows, “+” indicates positively correlated causations between system variables and “–” indicates negative correlated causations.

Figure 1 shows the major feedback loop that drives ecosystem decay: with each disappearance of a species from the system, all stabilising feedback loops in which this species were previously involved are lost. Even significant population declines weaken these feedback loops, promoting the chances of later extinction events. A decreased stability of ecosystems may then, in consequence, result in larger fluctuations in response to species loss, occasionally pushing more species towards extinction, forming a vicious cycle. In a fragile ecosystem, intrinsic oscillations or external disturbances are more likely to drive a species towards extinction or diminish its population size, which in turn will reduce the biomass in the ecosystem and decrease the intraspecific diversity. With lower population size, this leads to fewer and also to less diverse intraspecific interactions (i.e., interactions between individuals of the same species) and thus reduces the effect of existing feedback loops, which are mainly stabilising feedback loops in ecosystems that were previously resilient and robust. As a consequence, the resilience and stability of the system will be reduced, which in turn amplifies future amplitudes of population disturbances and fluctuations.

## Potential Ecological Effects of Robot–Organism Interactions

Technology, and in particular robotics, can offer open-loop solutions to better monitor, and also act on, threatened ecosystems (Grémillet et al., 2012). The approach we are proposing to counteract the observed ecosystem decay proactively is to use autonomous robots to be integrated into existing organism groups in a threatened ecosystem. This has to be done in a way that robots can interact as naturally as possible with their organismic counterparts. Every ecosystem contains species with a very high number of interspecific interactions (i.e., interactions with other species); these species are called “keystone species” (Power et al., 1996). Logically, these species are the number one candidates to interact with, as modulating their behaviour will have the maximum effect on the ecosystem they reside in. Figure 1 shows how autonomous robots can play a significant role in the vicious cycle of ecosystem decay. The robots can, on the one hand, proactively monitor the ecosystem by collecting data from within organism communities in which they are embedded and can alert human operators (blue boxes in Figure 1). Robots for proactive intervention, on the other hand, are designed in a way such that they can additionally interact with a specific organism group (orange boxes in Figure 1). They have to be able to perceive stimuli emitted by their organismic counterparts, to compute a sufficiently complex behavioural response and then to execute this response with appropriate actuators. These stimuli, sent by robotic actuators, are perceived by the living organisms and those will, in turn, respond to these stimuli in a desired way, e.g., by showing a desired behaviour or by modulating an already-performed behaviour. Such agents can often be bio-mimetic and mirror the living organisms they interact with; thus, they try to appear as a conspecific interaction partner by the focal organism. However, they can also in principle mimic any other organism that has an ecological relationship to the relevant organism, such as predators, prey, inter-specific competitors, and parasites or symbionts. We would like to point out that some approaches that would possibly work might cause ethical questions, for example, if a robot mimics a predator in order to have a repellent effect. Consequently, we exclude such approaches from our further considerations, as we restrict ourselves to technologies that do not increase the stress levels of organisms above the level of their regular, natural life. We also refrain from inducing stress from pain, threats or other severe negative emotional states of organisms with high cognitive capabilities.

So what is the most effective way to integrate robots into natural ecosystems? Population density is a key variable in ecological relationships, as interaction patterns depend in a super-linear way on the density of the interacting organism groups, following the “mass action law.” Uneven dispersal further affects the dynamics that arise from heterogeneous density distributions across the habitat. Thus, first monitoring and then potentially inducing a modulation of local densities can regulate key aspects of ecosystem dynamics. For example, the “competitive exclusion principle” (also known as the “Gause law”) describes processes that are strongly affected by interaction densities and the altered resource-sharing levels that arise when animals are unevenly distributed (Hardin, 1960). Ultimately, these processes are at the heart of explaining biological diversity (or lack thereof) and the ongoing niche construction and speciation that it is associated with.

**Our key hypothesis:** Technological artefacts, e.g., autonomous robots, can integrate into organismic populations and animal societies, in order to modulate their key processes, such as locomotion in animals and growth in plants. These modulations can affect the organisms in a way that alters their local population densities, which then can have significant ecological and social effects. We hypothesise that it is possible to design these technological agents in a way that they do not control the organisms by force, but rather become a part of the closed-loop control that governs the collective organismic system, bringing information into the regulation of the system that can be collected by technological means and can be useful to the organisms. This way, they can use very subtle stimuli in the microscopic and proximate interaction patterns in order to achieve a significant ultimate effect on the macroscopic ecosystem level.

**FIGURE 2:**
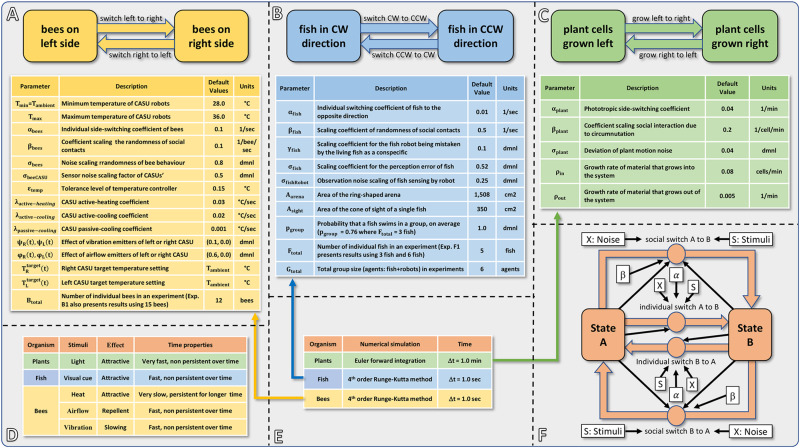
Summarising fact sheet of our models of bees, fish, plants and robots. **(A)** Basic structure and model parameters of the bee and robot model. **(B)** Basic structure and model parameters of the fish and robot model. **(C)** Basic structure and model parameters of the plant and robot model. **(D)** Overview of the modelled stimuli, the timing scale (how fast can they be emitted, how fast can they be removed from the system, and how persistent do they stay in the environment?) and the reaction they trigger. **(E)** Overview of the used numerical solver method, time step size, and used dimensions of time. **(F)** Commonalities of the models: overview showing the basic concept of all three modelling approaches with a social component and an individual component, indicating which parameters and variables affect which of these processes.

To provide a detailed illustration of how our hypothesised application of robotic actors can modulate key processes in organismic populations, we develop models for three specific bio-hybrid systems and show how they predict empirically obtained results. Importantly, the models that we develop share a common form, revolving around individual and socially mediated dynamics in each of the systems. As is extremely common in behavioural sciences, the assays considered here are formulated as a binary choice for the organisms. This provides clearly measurable outcomes in the behaviours and additionally enables the development of models that feature common elements. Before the detailed presentation of each model in sections “Honeybee and Robot Experimentation,” “Fish and Robot Experimentation,” and “Plant and Robot Experimentation,” we here provide an overview of their commonalities and differences. In each case, the organisms can choose to adopt one or other state, and the dynamics involve switching their choice. A switch can be mediated by a collective social influence, or by individual preference. The collective result of these two “forces” can lead to different dynamics such as even distributions or biassed distributions (including strong symmetry breaking). Even though the organisms that our robotic devices interact with are dissimilar (e.g., in motion speed, scale, and typical group size), a similar modelling approach is able to capture the dynamics in all three systems. Figure 2 summarises the form of the three models and also provides the parameters used.

## Towards a Proactive Contingency: Organismic Augmentation

We have devised the concept of “organismic augmentation” as a leading paradigm in our research. This concept describes guiding principles for how to create autonomous robots that can interact with keystone species of high ecological importance. These robots are designed to blend into these organisms’ communities and to affect them from within the collective without causing a disturbance of the processes that usually determine the behaviours of these agents. This can be achieved by bio-mimicking conspecifics (shown with fish here) or by altering the local environment of the organisms in a way that will also happen under favourable environmental conditions (shown with honeybees and plants here).

Our studies, which we present here, focus on a few examples of specific keystone species groups, which we think are of high ecological significance. Their well-being is also highly relevant for our human society:

(1) Honeybees, as they are the pollinators of plants, and thus facilitate plant growth and dispersal. Their foraging success is also a good indicator for a healthy ecosystem concerning flowering plants.
(2) Fish, as they are keystone aquatic species, and water covers about 71% of the earth’s surface. Fish are also a major food source for humanity.
(3) Vascular plants, as they are the trophic basis of ecosystems, serving as food and as a shelter place for many animals and also feed humanity.

Social organisms already have a natural “interaction interface” that is provided by their social interaction patterns. Therefore, we suggest that integrating autonomous robots into social animal communities may be the most promising approach to achieve animal–robot interaction. Thus, as an easy approach towards robot–animal integration, robots should be able to take part within the social interaction networks of their target organisms. The fact that many social animals are also keystone species in their ecosystems increases the significance of this social interaction approach. For example, honeybees and bumblebees are major pollinators, together with wasps, which are also major predators. Ants facilitate the destruction of organic materials, but also act in seed dispersal and as symbionts of aphids, which in turn interact as strongly aggregated communities with plants.

**FIGURE 3:**
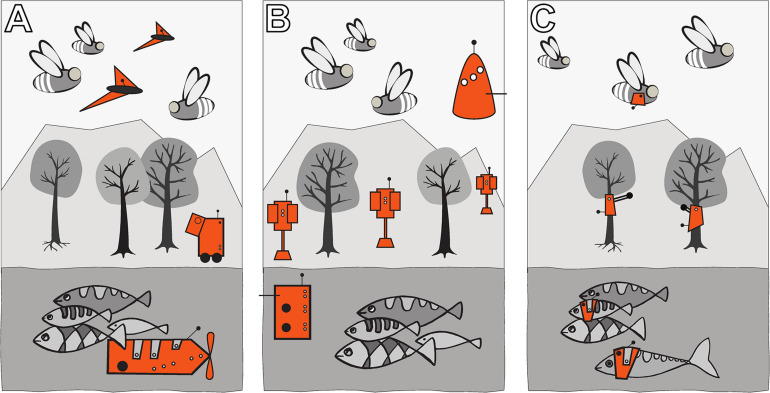
Augmentation of organismic populations may be implemented in three main forms (Mondada et al., 2013). **(A)** By introducing mobile devices into the ecosystem. These agents are able to interact with the natural organisms using specifically designed stimuli. **(B)** By adding fixed devices in the environment. These devices exhibit agency and can create environmental conditions that have an impact on the ecosystem and specifically on the organisms that are addressed with the system. **(C)** By mounting devices directly on the individuals and impacting their behaviour by an interaction that takes place directly on their body. This way, the animals become biohybrid agents themselves.

Autonomous robots can be designed in three ways to achieve a “guided locomotion” functionality, as it is suggested by Mondada et al. (2013) and Halloy et al. (2013); see Figure 3.

First, they can be mobile agents that locomote together with the organisms, for example, in group motion patterns; see Figure 3A. The way of locomotion does not necessarily have to be identical to the locomotion of the organisms, as long as it does not disturb them in any way. Various approaches along these lines have been performed with fish robots, either with magnetic coupling or mounted on a rod (Faria et al., 2010; Donati et al., 2016; Landgraf et al., 2016; Bonnet et al., 2017b; Worm et al., 2017; Porfiri et al., 2019; Romano et al., 2019; Utter and Brown, 2020), with wheeled robots interacting with cockroach communities (Halloy et al., 2007) or flocks of ducks (Vaughan et al., 2000) and with a dancing robot with honeybee foragers (Landgraf et al., 2010). In all these cases, the locomotion of the robot was achieved differently from the locomotion of the living animal counterparts, and the robots were of varying bio-mimetic perfection, some just emitting the key stimuli necessary for influencing the organisms (Tinbergen, 1951).

Second, the robots may be distributed as an array of sensor–actuator nodes that can sense and locally act, but do not themselves locomote; see Figure 3B. We call such sensor–actuator nodes combined actuator sensor units (CASUs), as they are described in Schmickl et al. (2013) and Griparić et al. (2017). Experiments with static arrays of CASUs were performed by modulating honeybee aggregations (e.g., Stefanec et al., 2017a; Mariano et al., 2018) and by guiding plant growth (Wahby et al., 2018). In such a static array, the agents themselves cannot move, but they can emit stimulus patterns that show spatio-temporal dynamics, sometimes produced by nearest-neighbour interactions of adjacent robots in the topology, similar to how cells do in cellular automata (Wolfram, 1983). It is possible that the array reconfigures itself slowly over time, similar to the array/network of under-actuated mobile units described in Donati et al. (2017) and Thenius et al. (2018), which are primarily aimed at long-term environmental monitoring but can act as a CASU with the appropriate organisms as well. For example, such long-term interactions with organisms are explored (Heinrich et al., 2019) for the prospect of creating adaptive and self-healing living architecture.

Third, guided locomotion can be achieved by technically augmenting single individuals by mounting autonomous devices onto living organisms in order to influence their behaviours and ultimately guide the whole social group (Butler et al., 2006; Tsang et al., 2010); see Figure 3C. This approach can raise ethical concerns, especially if social higher vertebrates are used; thus, we are not further considering this approach here. In our approach, we are not mounting devices on single individuals but integrate devices into social organism societies to influence the organismic groups from within (see Figure 3B).

**FIGURE 4:**
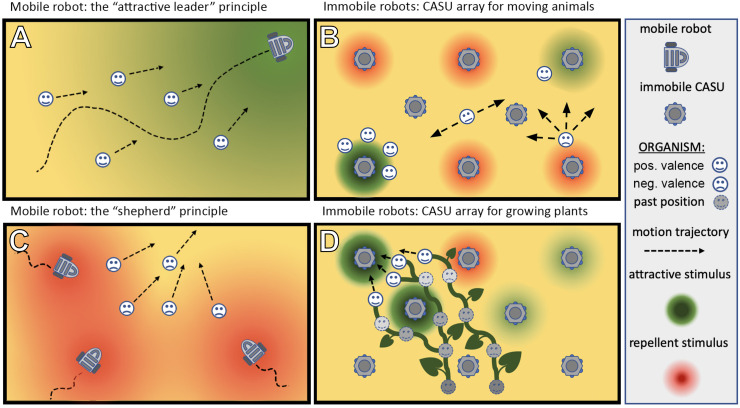
Different types of set-up in which robots can be used to interact with living organisms. **(A)** A mobile robot can lead the organisms by emitting an attractive stimulus/exhibiting an attractive behaviour. **(C)** A mobile robot can herd the organisms in a desired direction by emitting a repellent stimulus. **(B,D)** An array of sensor–actuator nodes (CASUs) can exhibit patterns (either in time or space or both simultaneously) of repellent and/or attractive stimuli to guide organisms [animals **(B)** or plants **(D)**] to a desired place or in a desired direction.

The ways in which autonomous robots can interact with organisms are manyfold: for example, they may take a leader role and guide the organisms in their locomotion behaviour, e.g., with swarming, flocking, herding, shoaling, and schooling animals (Figure 4A). In case the target organisms are plants, the robots could guide them in their growth (Figure 4D). In these cases of “guided locomotion,” the organisms may be directly led away from unfavourable or even dangerous places (pollutants, over-harvesting, predation, hot spots of pests, etc.) and guided towards more favourable places. Besides direct guidance by the robots, it is also possible for robots to just give a subtle bias to the organism motion, e.g., by locally modulating environmental cues (e.g., light and temperature), and to exploit specific locomotion strategies of organisms this way (Figure 4B). Such strategies might include Levy walks/flight (Viswanathan et al., 2008), klinotaxis (Izquierdo and Lockery, 2010), and coordinated group motion (Herbert-Read, 2016). Organisms often perform such motion principles in nature; and even a subtle modulation of specific environmental factors or of specific interaction patterns can nonetheless lead to significant changes in the overall long-term motion of such organisms.

Besides the guided motion, robots could also affect the dispersion properties of populations, which can range from strong avoidance (Figure 4C), like in territoriality (low intra-specific contact rates), over diffusion-like random dispersal (medium intra-specific contact rates) to aggregation behaviours (high intra-specific contact rates). Thus, “guided dispersal” and “guided aggregation” strategies performed by autonomous robots can significantly affect important ecological variables. For example, the frequency of intra-specific interactions affects critical aspects of all life forms that we know:

(a) Intra-specific competition imposes the most important negative feedback loop that keeps populations in balance under natural conditions and the main driving force for natural selection and thus for biological evolution.
(b) For sexually reproducing organisms, mate finding is a vital aspect for reproduction, as too low a population density can impair the success rate of finding mates for reproduction. This was shown to be the final nail in the coffin of some sexually reproducing species’ populations, a fact that is known as the “Allee effect” in ecology (Stephens and Sutherland, 1999).
(c) Effects of high population densities, as they occur in aggregations, can be “negative” ones for population dynamics, e.g., parasite pressure and infection rates, but “positive” effects can also occur, e.g., induced by symbionts, or information spread in the case of communicating organisms.

All these important biological aspects can be modulated by changing the dispersal patterns of organisms in their environment. Appropriately designed robots can interact with animals in a way that these motion patterns and their ultimate dispersal effects can be influenced.

Depending on their design, robots can impact aspects other than the spatial organisation of members of the society. They can collaborate with the individuals of the society on specific tasks, like foraging, waste removal and control of nest conditions. Thus, such robots can affect ecological aspects or organisms and, ultimately, can affect the whole ecosystem in which these organisms participate.

In order to induce behavioural changes, especially for the “guided dispersal” and “guided aggregation” functionalities, the autonomous robots need to be able to perform a richer “vocabulary” than just emitting attractive signals. To be able to exert control over the organisms’ spatial dispersal patterns, a set of stimuli has to be found that (a) the robot can emit and (b) the organism reacts to. For ethical reasons, we restrict ourselves here to stimuli that are (i) naturally occurring in the organism’s natural environment at a sufficiently regular rate and (ii) emitted in a strength that is also in the naturally occurring spectrum, and (iii) which have no known negative side effects on the organisms.

We identified the following three basic signals or cues that are required to have sufficient effect and control of the organisms’ dispersal patterns:

(A) *Attractive stimulus*: This stimulus should be attractive for the animals and lead to aggregations over time around the places it is emitted. This can be a direct effect on gradient-exploiting individuals (tropotaxis) or a modulation of turning probabilities (e.g., in klinotaxis) or modulation of social interaction (grouping) behaviours. Basically, it can be translated into “Come here!”
(B) *Repellent stimulus*: This stimulus is the inverse of the aggregating stimulus, operating along the same mechanisms as mentioned above, however, acting in the opposite direction. It basically means “Go away!”
(C) *Speed modulating stimulus*: This stimulus should be able to modulate the speed of animals, or the growth rate of plants. In an extreme case, it should be able to stop any motion, basically meaning “Stay where you are!”

**FIGURE 5:**
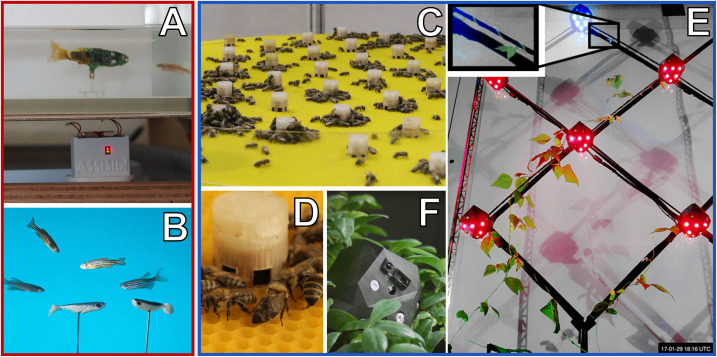
Examples of mobile robots (red frame) and immobile artefacts (blue frame) that can interact with animals or plants by emitting various stimuli. **(A)** Free-moving fish robot with an active (tail-beating) lure that was developed in the project ASSISI_bf for interacting with zebrafish. **(B)** Close-up of a mixed swarm of fish robots (only coupled lures visible) and zebrafish. **(C)** Horizontal array of combined actuator sensor units (CASUs) that was developed in the project ASSISI_bf for interacting with honeybees. **(D)** Close-up of one CASU surrounded by honeybees. **(E)** Vertical array of CASUs, developed in the project *flora robotica* to guide plant growth; inset frame shows a plant tip approaching the top-most robot (Figure “*Main result; predefined-pattern experiment*”: from Wahby et al., 2018, licenced under CC BY 4.0; colours modified). **(F)** Close-up of a CASU to guide plant growth, surrounded by plants.

These stimuli can have arbitrary shapes (e.g., binary on/off signals, continuous cues or even a combination of both) that are spread around the robots’ local environment. In addition, these stimuli can be physically similar (vision/light, vibration/sound, smell/taste, touch, etc.), meaning that the receiving organisms use the same receptor types to perceive them but still react differently. In the case of similar stimuli inducing different behaviours in the organisms, the specific “meanings” of each signal have to be encoded in its characteristics (e.g., waveform shape, amplitude, and frequency). This is not something that can be designed arbitrarily, because it is the organisms who determine which stimuli they react to; therefore, these control stimuli have to be identified by sufficiently observing and analysing the animal’s behaviour and interactions before designing the robots. However, it might also be that these three stimuli/signals/cues (A, B, and C) all reside on very different physical channels. This latter approach has the significant advantage that multiple stimuli can be emitted in parallel and, if designed correctly, with no, or negligible, interference. On the downside, stimuli emitted through different physical channels usually have very different timescales on which they can be changed in the environment; e.g., a light signal propagates quickly in contrast to a temperature change that propagates and decays much more slowly. In our framework, we call an autonomously and free moving agent a “robot” (Figure 5A) and groups of such agents a “robot(ic) swarm” (Figure 5B). In contrast to that, we call technological artefacts that cannot move a CASU (Figures 5D,F) and to a spatially distributed collection of these agents as a “CASU array” (Figures 5C,E).

In order to be efficient and effective, but also ethically correct, one has to understand the organism system first before designing the robots to be introduced into the specific community. It is also important to understand the collective biohybrid system that is created by introducing the robots. Therefore, we here focus on presenting mathematical models and simulations of animal–robot and plant–robot systems that were created under lab conditions. While some work on the robotic and experimental side of these systems has been published, there is a lack of a general understanding of these systems, of their commonalities and of their specific elements. Such a more general understanding of the system not only can inform future engineers of similar or other biohybrid systems but also can allow us to understand the physically established system in a more general way, which is an important step to leave the lab behind and to employ these understandings into technical artefacts that unfold their potential with living organisms in the wild.

Many robot–organism interaction systems are still in a “lab only” phase, for example, when magnetic coupling through a fish tank’s glass wall or rods from above are used to drive fish-mimicking robots. While these set-ups can be very valuable for basic research of individual and collective behaviours *per se*, there is no way to implement such robots in the wild. For application in the field (pond, lake, river, and ocean), the locomotion methods would need to be changed, for example, into an undulating robot fish (Kruusmaa et al., 2014). Other technologies, like the approach to put non-mobile robots such as a CASU array into the environment, are already closer to being implemented outside of the lab. Thus, in section “The Next Step: Leaving the Lab and Bringing the Robots Into the Wild,” we will showcase how the understanding of the honeybee-and-robot system in the lab experiments was converted into simpler devices that can affect full honeybee colonies in the natural environment, where they act as important pollinators and thus such systems could be utilised as a distributed long-term and wide-range stabiliser and supporter of ecosystems in which these bees play an important role.

### Honeybee and Robot Experimentation

**FIGURE 6:**
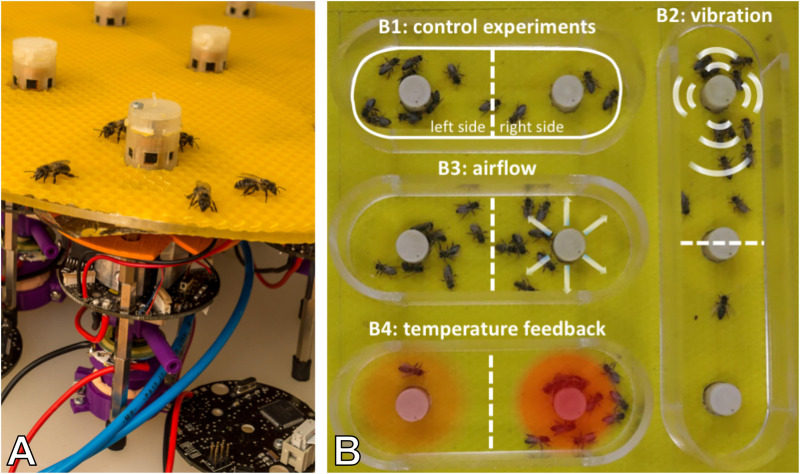
Combined actuator sensor unit (CASU) for bees developed in the project ASSISI_bf and experimental set-ups. **(A)** CASUs with surrounding honeybees: above the arena floor, which is covered with beeswax sheets, is the cylindrical top part that houses the six infrared sensors for bee detection (sensing radius approximately 2 cm) and the airflow nozzles. Below the arena floor is the bottom part of the CASU with the heat-exchange and vibration devices and the air pipes (single-board computers connected to the CASUs not shown). **(B)** Experimental set-up for testing (B1) the natural symmetry breaking in collective decision making of bees in constant temperature fields, (B2) symmetry breaking in collective decision making induced by vibration, (B3) collective decision making modulated by airflows, and (B4) the effect of robot-induced feedbacks on the symmetry breaking in collective decision making. Solid white line represents the evaluation area for counting the bees, divided by the dashed line (left side and right side).

To investigate the capability of immobile robots to interact with honeybees, we performed a set of experiments in which the robots altered the local environment by exhibiting various stimuli. The aim was to measure the influence of the different “communication channels” of the robots on the animals’ aggregation behaviour (i.e., spatial distribution). The robotic nodes, called CASUs, used in these experiments were developed specifically to integrate themselves in groups of young honeybees by (i) being able to sense nearby bees and (ii) having the ability to exhibit the appropriate signals (as defined in section “Towards A Proactive Contingency: Organismic Augmentation”) to effectively affect young bees, namely, (a) temperature as an attractive stimulus, (b) vibration as a speed-modulating stimulus, and (c) airflow as a repellent stimulus (see Figure 6).

All these stimuli are ubiquitous in a normal honeybee hive (e.g., thermoregulation of the brood nest, various vibrational communication signals, and wing fanning to produce air circulation); and the stimulus intensity that the robots could apply was within the range naturally occurring in the beehive; i.e., no abnormal stimulus was applied to guide the animals during interaction with the robotic nodes.

We identified the aggregation behaviour of freshly emerged bees as a suitable test case to study organismic augmentation in honeybees because (i) the group behaviour is influenced by local environmental conditions (e.g., temperature) and (ii) simple cues could be identified to govern the aggregation behaviour (e.g., bees’ stopping times after contact with a conspecific) (Szopek et al., 2013), both of which can be exploited by the CASUs to affect the bees’ behaviour.

#### Animals

All experiments with honeybees (*Apis mellifera* L.) were performed at the Department of Biology at the Karl-Franzens-University Graz, with young bees, aged from 1 to 24 h. At this age, the bees are not yet able to endothermically produce heat with their wing muscles (Stabentheiner et al., 2010), nor are they yet able to fly or sting. To collect the bees, sealed brood combs were removed from full colonies and incubated at 35°C and 60% relative humidity. After hatching, the freshly emerged bees were brushed off the combs and housed in a ventilated box on a heating plate at 35°C and fed honey *ad libitum* before and after the experiments. Each bee was only tested once, and all bees were introduced into full colonies at the end of the day.

#### Robotic Combined Actuator Sensor Unit Array Arena

The experimental set-up consisted of a horizontal surface equipped with an array of robotic nodes that were specifically developed to integrate into groups of young honeybees (see Figures 5C,D, 6). Each robotic node was equipped with six infrared sensors to detect the surrounding bees, and temperature sensors and actuators to generate stimuli that bees are reacting to, including temperature, vibration, and airflow. The robots were controlled by Beaglebone single-board computers, which also executed the user-level controller, facilitated communication with other robots and the host PC, and provided data logging.

For the specific experiments discussed here, only a subset of robotic nodes was used with either two or three CASUs that were enclosed by a stadium-shaped Plexiglas arena to keep the bees within a certain area around them (see Figure 6B).

Above the top part of the robot, the arena floor was covered in beeswax sheets that were replaced after each repetition to get rid of any possible odour remnants that could interfere with the bees’ behaviour. All experiments were performed in IR lighting conditions with wavelengths above the bees’ sensitivity to exclude any visual stimuli and captured with a camera sensitive to IR light (Basler ac2040-25gmNIR) mounted above the arena. For a detailed description of the system, see Griparić et al. (2017).

#### The Model of Robots and Bees

The minimal model arena is composed of two sides, each containing a CASU. The dynamics of the CASUs controlling the local temperatures of each side of the arena and the number of bees on each side are modelled. In the following, the temperatures of the arena’s right and left sides are represented by *T_R_*(*t*) and *T*_*L*_(*t*), respectively. These temperatures are modulated by the CASUs located on the two sides, which either set the local environment to a fixed temperature or set the temperature according to the locally sensed numbers of bees.

The number of bees on the right and left sides are represented by *B*_*R*_(*t*) and *B*_*L*_(*t*), respectively, whereby *B*_*R*_(*t*)+*B*_*L*_(*t*) = *B*_total_. Initially, they are assumed to be symmetrically split up between the two sides; thus, *B*_*R*_(0) = *B*_*L*_(0) = 0.5⋅*B*_total_ each. In our model, we assume that all bees move randomly and stop at bee–bee encounters and that the duration of the resting of bees after such collisions depends on the local temperature (Szopek et al., 2013), while the average speed of the bees can be affected by ground vibrations (Mariano et al., 2018). In addition, we show here that a subtle airflow can also affect the bees’ behaviour by reducing their resting time after social interactions. Therefore, these three stimuli affect the rates of change of honeybee aggregations that form around stimuli-emitting robots. Bees that leave one cluster run randomly and eventually re-join the same cluster or join a cluster around another robotic CASU. Our model is based on depicting the dynamics of bee aggregations resulting from the robot-induced modulations of these rates of change.

The overall changes in the number of bees on each side are computed by two ordinary differential equations (ODEs) (Eqs B-1a,b) that describe the changes of bees on the left and right arena sides, by balancing the flows of bees modelled in Eqs B-2a,b and B-3a,b, as

(B-1a) $$\begin{array}{c} \frac{dB_{R}}{dt}={\text{switch}}_{R}^{\text{indiv}}\left(t\right)-{\text{switch}}_{L}^{\text{indiv}}\left(t\right)\\ \;+{\text{switch}}_{R}^{\text{social}}\left(t\right)-{\text{switch}}_{L}^{\text{social}}\left(t\right), \end{array}$$

(B-1b) $$\begin{array}{c} \frac{dB_{L}}{dt}={\text{switch}}_{L}^{\text{indiv}}\left(t\right)-{\text{switch}}_{R}^{\text{indiv}}\left(t\right)\\ \;+{\text{switch}}_{L}^{\text{social}}\left(t\right)-{\text{switch}}_{R}^{\text{social}}\left(t\right). \end{array}$$

Those bees that are not resting on each side may move to the other side due to their random movement in a diffusion-like process, which can be nicely modelled with a mean-field approach, e.g., by systems of ODEs. A cluster of bees around one robot may grow in two different ways:

*Individual side switching:* On the one hand, a cluster on the ipsilateral side can grow from bees joining after having left the contralateral CASU area and, after traversing the arena, spontaneously stop without any social interaction. Consequently, this process does not depend (scale) on the number of bees that are already present at the ipsilateral side, but it will change in proportion to the bees leaving the contralateral side. The stopping probability at which this happens is expressed by the constant α_bees_, which regulates the rate at which this individual spontaneous stopping happens, while the variables τ_*R*_(*t*) and τ_*L*_(*t*) represent the resting times that bees exhibit on either side depending on the local temperature they encounter there. The individual stopping flows can thus be modelled as

(B-2a) $${\text{switch}}_{R}^{\text{indiv}}\left(t\right)=\alpha_{\text{bees}}\cdot X_{R}^{\text{indiv}}\left(t\right)\cdot \frac{B_{L}\left(t\right)}{\tau_{L}\left(t\right)},$$

(B-2b) $${\text{switch}}_{L}^{\text{indiv}}\left(t\right)=\alpha_{\text{bees}}\cdot X_{L}^{\text{indiv}}\left(t\right)\cdot \frac{B_{R}\left(t\right)}{\tau_{R}\left(t\right)},$$

where $X_{R}^{\text{indiv}}\left(t\right)∼U\left(1-\sigma_{\text{bees}},1+\sigma_{\text{bees}}\right)$ and $X_{L}^{\text{indiv}}\left(t\right)∼U\left(1-\sigma_{\text{bees}},1+\sigma_{\text{bees}}\right)$ are the scaled noise functions; the parameter σ_bees_ ∈ [0,1] scales the noise. Equation B-2a expresses that in each time step *t*, a number *B*_*R*_(*t*)/τ_*R*_(*t*) of bees will leave the cluster on the right side and with a probability of α_bees_ they will stop and thus join the cluster on the left side of the arena (and similarly for bees leaving the left side in Eq. B-2b). Thus, the number of moving bees that can stop on one (ipsilateral) side is the inverse of the waiting time of the bees on the other side $\left(\frac{1}{\tau_{L}\left(t\right)}\;\text{and}\;\frac{1}{\tau_{R}\left(t\right)}\right)$.

*Socially induced side switching:* On the other hand, bees may also leave their cluster on the contralateral side and accidentally meet with bees on the ipsilateral side in their random walk and, consequently, join the ipsilateral cluster as a socially induced event. Again, this switching is inversely related to the bees’ waiting time at their place of origin, which in this case is from the contralateral arena side. It is additionally proportional to the number of bees already present at the ipsilateral side, following the concept of mass action law, which is often used in modelling biological interactions, e.g., in predation, competition, or infection models. A parameter β_bees_ is used here to model the rate of the social contacts, which are a consequence of the random walk behaviour that bees often exhibit.

(B-3a) $${\text{switch}}_{R}^{\text{social}}\left(t\right)=\beta_{\text{bees}}\cdot X_{R}^{\text{social}}\left(t\right)\cdot B_{R}\left(t\right)\cdot \frac{B_{L}\left(t\right)}{\tau_{L}\left(t\right)},$$

(B-3b) $${\text{switch}}_{L}^{\text{social}}\left(t\right)=\beta_{\text{bees}}\cdot X_{L}^{\text{social}}\left(t\right)\cdot B_{L}\left(t\right)\cdot \frac{B_{R}\left(t\right)}{\tau_{R}\left(t\right)},$$

where $X_{R}^{\text{social}}\left(t\right)∼U\left(1-\sigma_{\text{bees}},1+\sigma_{\text{bees}}\right)$ and $X_{L}^{\text{social}}\left(t\right)∼U\left(1-\sigma_{\text{bees}},1+\sigma_{\text{bees}}\right)$ are the scaled noise functions, the parameter σ_bees_ ∈ [0,1] scales the noise, and the parameter β_bees_ is a coefficient modulating the strength of the social interaction process that leads to cluster formation. By adjusting the ratio $\frac{\alpha_{\text{bees}}}{\beta_{\text{bees}}}$, the specific contribution of individual and social stopping behaviours to the cluster formation process can be adjusted in this system.

The model is driven by the diffusion of bees in the arena and by the modulated durations of the resting time, after they stopped either individually or socially. These resting times can be modulated by three types of stimuli that can be emitted by the robots, and which affect the bees in different ways, as is incorporated in the model in the remainder of this section.

As the most prominent behaviour-modulating stimulus is temperature, we model the effect of temperature on the bees’ behaviours to a larger extent than the other stimuli. This is also necessary because the thermal stimulus influences the environment for longer periods than the other types of used stimuli and thus requires a specific submodel. It was found that young honeybees move mostly randomly when they walk in temperature fields that are similar to the thermal conditions in a beehive and stay for some time at the place after they “bumped” into other bees (Kernbach et al., 2009; Szopek et al., 2013). The mean resting time duration after such bee-to-bee contacts was found to follow a sigmoid-shaped function of the local temperature at the place of the encounter. As both robotic CASUs modulate the local temperature in their vicinity, we model the bees’ waiting times separately for each side by using a hill function, taking the local temperatures (*T*_*L*_(*t*) for the local temperature in the left half of the arena and *T*_*R*_(*t*) for the right side) as their only input.

(B-4a) $$\begin{array}{c} \tau_{R}\left(t\right)=\left(1+\frac{\tau_{\text{Δ}}}{T_{\text{Δ}}}\cdot \left(T_{R}\left(t\right)-T_{\text{min}}\right)\cdot \left(1-\varphi_{R}\left(t\right)\right)\right)\cdot \\ \;\left(1+\psi_{R}\left(t\right)\right), \end{array}$$

(B-4b) $$\begin{array}{c} \tau_{L}\left(t\right)=\left(1+\frac{\tau_{\text{Δ}}}{T_{\text{Δ}}}\cdot \left(T_{L}\left(t\right)-T_{\text{min}}\right)\cdot \left(1-\varphi_{L}\left(t\right)\right)\right)\cdot \\ \;\left(1+\psi_{L}\left(t\right)\right), \end{array}$$

where τ_*R*_(*t*) and τ_*L*_(*t*) are the resting time periods of the bees at the right and left sides of the arena, respectively, using a linear function of the local temperature that approximates the sigmoid previously used to fit empirical data: The waiting time is 1.0 s for a temperature of 28.0°C (our minimum ambient temperature) and scales linearly for a range τ_Δ_ = 24.0s over a span of T_Δ_ = 8.0°C of temperature increase, as we observed a waiting period of 25 s with bees at 36°C (which is the highest temperature used in our experiments) in Mills et al. (2015). The honeybees’ resting behaviour is also influenced by vibration and airflows, factors that are also considered in Eqs 4a,b. The variables φ_*L*_(*t*),φ_*R*_(*t*) ∈ [0,1] represent the effect of a subtle airflow emitted by the left or right CASU, acting as a repellent stimulus and inducing a shortening of the bees’ resting periods around these robots. In contrast, the variables ψ_*L*_(*t*),ψ_*R*_(*t*) ∈ [0,1] represent the effect of ground-carried vibration, emitted by the left or right CASU, acting as a speed-reducing or even stopping stimulus, thus inducing an increase of the bees’ resting periods around these robots.

The robotic CASUs in our system have their own agency, which needs to be part of the model that should depict the overall biohybrid system. Our honeybee CASUs have sensors to detect the bees in their vicinity. The CASU actively regulates the temperature based on the number of locally detected bees, if this regulation is enabled. We assume that the CASUs detect the bees in an imperfect way, as there are several “blind spots” and also a limited sensor range around these robots. We modelled the honeybee detection as follows.

For each CASU, there is a given target temperature towards which it is actively controlling its local environment: $T_{L}^{\text{target}}\left(t\right)$ for the left CASU and $T_{R}^{\text{target}}\left(t\right)$ for the right CASU. These target temperatures can (a) be preset to constant values, or (b) follow pre-programmed time patterns or (c) be set dynamically by the CASU’s control program in response to sensing bees with its IR sensors in its vicinity. In cases (b) and (c), a fixed-step incremental controller is used to model the heating and cooling that drive the actual temperature around CASUs towards the given target temperatures. If the actual temperature is further below the target temperatures than a given threshold ε_temp_, then the CASU will heat with a fixed rate λ_heating_ towards the target. Similarly, if the actual temperature is further above the target temperature than ε_temp_, the CASU will cool with a fixed rate λ_cooling_ towards the target. Finally, passive diffusion is modelled as proportional to the difference between each CASU and the ambient temperature Tambient=28C∘, with coefficient λ_passive_. These factors together yield the following equations:

(B-5a) $$\begin{array}{c} \frac{dT_{R}}{dt}=-\lambda_{\mathrm{passive}-\mathrm{cooling}}\cdot \left(T_{R}\left(t\right)-T_{\text{ambient}}\right)\\ \;+\left\{ \begin{array}{cc} \lambda_{\mathrm{active}-\mathrm{heating}}\cdots \text{if}\;\left(T_{R}^{\text{target}}\left(t\right)-T_{R}\left(t\right)\right)>\varepsilon_{\text{temp}} & \\ -\lambda_{\mathrm{active}-\mathrm{cooling}}\cdots \text{if}\;\left(T_{R}\left(t\right)-T_{R}^{\text{target}}\left(t\right)\right)>\varepsilon_{\text{temp}} & \end{array}\right., \end{array}$$

(B-5b) $$\begin{array}{c} \frac{dT_{L}}{dt}=-\lambda_{\mathrm{passive}-\mathrm{cooling}}\cdot \left(T_{L}\left(t\right)-T_{\text{ambient}}\right)\\ \;+\left\{ \begin{array}{cc} \lambda_{\mathrm{active}-\mathrm{heating}}\cdots \text{if}\;\left(T_{L}^{\text{target}}\left(t\right)-T_{L}\left(t\right)\right)>\varepsilon_{\text{temp}} & \\ -\lambda_{\mathrm{active}-\mathrm{cooling}}\cdots \text{if}\;\left(T_{L}\left(t\right)-T_{L}^{\text{target}}\left(t\right)\right)>\varepsilon_{\text{temp}} & \end{array}\right., \end{array}$$

where *dT*_*R*_(*t*)/*dt* and *dT*_*R*_(*t*)/*dt* define the two ODEs that model the temperature changes around the left and right CASU areas, which feed into the waiting time curves of the bees that are defined in Eqs B-4a,b. Thus, in those cases that the target temperatures of CASUs are affected by the local number of bees, the system exhibits a closed-loop control between robotic CASUs and the honeybees.

For specific experiments with bees, specific settings, time patterns or control programs were used for the variables ψ_*R*_(*t*), ψ_*L*_(*t*), φ_*R*_(*t*), φ_*L*_(*t*), $T_{R}^{\text{target}}\left(t\right)$, and $T_{L}^{\text{target}}\left(t\right)$. These specific actuation regimes of heating, cooling, vibration, and airflow are described in the sections below, together with the corresponding experiments. Otherwise, the default values given in Figure 2A were used for these variables.

#### Experiments With Robots and Bees

In this section, we will detail the methodology for the four experimental sets that were performed with CASUs and honeybees. First, we establish a baseline of the natural collective behaviour of honeybees without active robotic agents. Second, we investigate how local vibration influences collective decision-making processes. Third, we investigate how robotic agents affect bees with a subtle airflow. Fourth, we investigate how honeybee decision making can be influenced by robots integrated in a closed loop producing warmth around them in reaction to higher bee densities. These empirical experiments validate our model of the biohybrid system, solved with Runge–Kutta fourth-order method with Δ*t* = 1.0s.

##### Experiment B1: Assessing the natural symmetry breaking in collective decision making of aggregating honeybees under non-time-varying temperature fields

To investigate the natural clustering behaviour of the bees in constant thermal environments, we performed experiments with groups of bees in a stadium-shaped arena with two CASUs set to fixed temperatures. We performed experiments in two settings: (1) Runs with 28°C on both arena sides were made with *N=14* repetitions for 20 min, containing groups of *B*_total_ = 12 bees that were released in the centre of the arena; and (2) runs with 32°C on one side of the arena and 36°C on the other side. This setting was tested *N=12* times for 13 min with *B*_total_ = 15 bees each. The target temperatures remained fixed throughout the runs, with no influence from the bees or the other CASUs.

**FIGURE 7:**
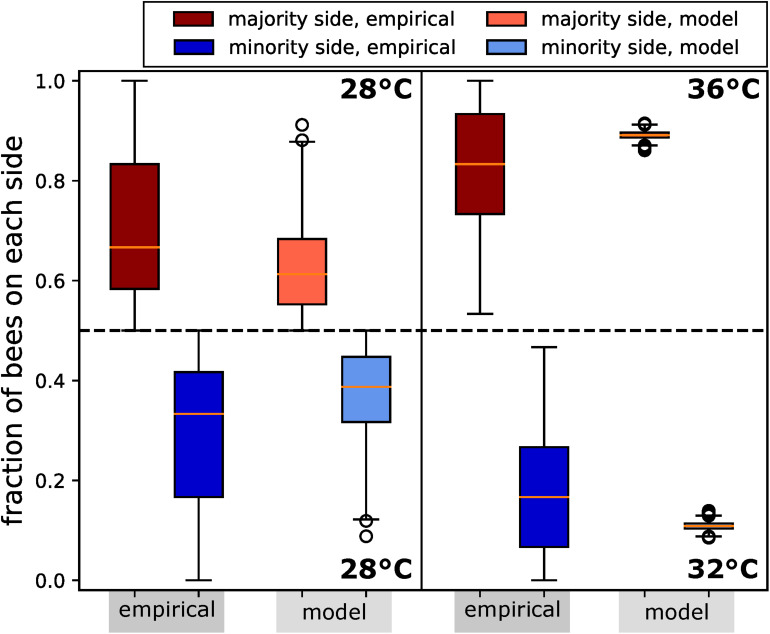
Honeybee group decision making in fixed environments, from empirical study and ordinary differential equation (ODE) model as described in the text. Two scenarios are considered: (1) a homogeneous environment, where the two choices are equal at 28°C, with *N=14* repetitions; (2) a heterogeneous environment, with one global optimum of 36°C and one local optimum of 32°C, with *N=12* repetitions. We measured the number of bees on the side with the majority for the period of 8–13 min. Since the group size differed between the two experimental settings, we report in fraction of the total group. We also display the distributions of fractions on the minority side. In setting (2), each bee group makes substantially stronger decisions than in setting (1), where there is no environmental difference to select on. Despite this, their social preference means that in setting (1), we still observe bees forming aggregations on one or another side to some degree. In both settings, the model generates a lower variance but otherwise predicts the aggregation effect corresponding to the empirical data.

In our analysis, we counted the bees on each side of the arena in 30-s intervals from video recordings, which were conducted under red-light conditions, to emulate the darkness of a beehive. For comparison, and to allow the bees an initial time to settle their collective decision making, we analysed the bees’ aggregations on both sides from minute 8 to minute 13 (Figure 7).

##### Experiment B2: Symmetry breaking in collective decision making induced by vibration

In this experiment (Mariano et al., 2018), a set of three CASUs aligned in a row were used, in contrast to the experiments described above, which used only two CASUs, in order to isolate the two arena sides better from ground-carried vibrations arriving from the other side. During the first 3 min, the bees could freely distribute themselves in the arena, as no vibration was produced by the CASUs; thus, ψ_active_(*t*) = ψ_passive_(*t*) = 0.0, for *t* ∈ [0,180]. Afterwards, the leftmost CASU started to emit a vibration pattern for another 3 min. The empirical study we validate our model against reports a set of vibration signals that were shaped by evolutionary computation algorithms to effectively slow down or even stop the bees. For *t* ∈ [181,360], we set ψ_active_(*t*) = 0.1 to model the effects of the vibration pattern spreading through the arena floor locally around this CASU on the bee behaviour. In contrast, the other CASU stayed passive, i.e., ψ_passive_(*t*) = 0, for *t* ∈ [181,360]. The parameter value ψ_active_ was chosen to fit empirical data.

We studied groups of *B*_total_ = 12 young (1 day old) honeybees in each arena in this experiment. In order to compare the reported empirical data in this setting in our mathematical model, we again consider the two sides of the arena—attributing the bees around the leftmost CASU area fully to the left side in the model in *B*_*L*_(*t*) and the bees around the rightmost CASU area to the right side of the model in *B*_*R*_(*t*)—and split the population of bees around the middle CASU 50:50 amongst the two model variables *B*_*L*_(*t*) and *B*_*R*_(*t*).

**FIGURE 8:**
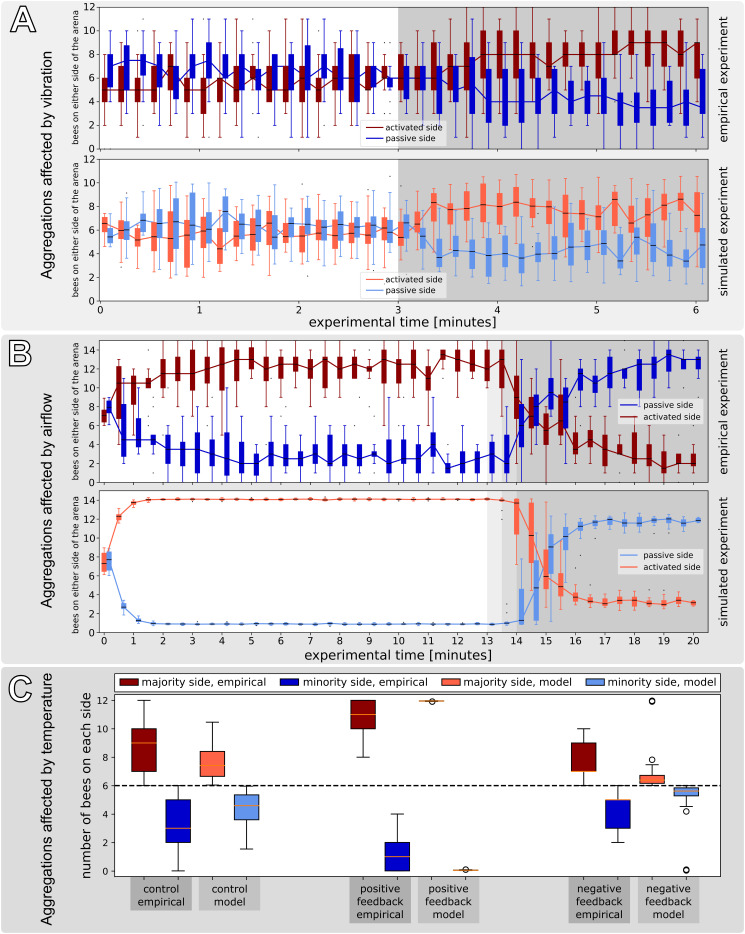
Effects of vibration, airflow stimulation and temperature on honeybee groups in empirical experiments and in our mathematical model. **(A)** Vibrational patterns were used to guide aggregation by moving the bees from an even distribution around the robots to an uneven distribution (*N* = 17 independent repetitions). The duration of the active vibration is indicated in the diagrams by the grey background: [ψ_active_(*t*) = 0.1 for *t* ∈ (181,360)]. In the first half of the experiment [ψ_active_(*t*) = 0 for *t* ∈ (0,180)], the bees move around freely and do not show any preference for one side of the arena. After the activation of the vibration (at time *t* = 181), there are more bees on the vibrating side in both the empirical experiments and the mathematical model. **(B)** In this experiment, an airflow stimulus was used to reverse initial decision making of honeybees in a temperature field containing a global optimum temperature (36°C at the “activated side” of the arena) and a local optimum (32°C, “passive side” of the arena), with *N* = 12 repetitions. The airflow was switched on at the robot on the warmer side to guide dispersal, which happened in the empirical experiments at different times between minute 13 and minute 15 as indicated by the grey background. This airflow stimulus remained active for the rest of the experiment. In the first phase of the experiment, more bees clustered around the warmer robot, while after activation of the airflow stimulus at this robot, bees increasingly dispersed and then aggregated around the other, cooler robot without airflow stimulus. These dynamics are replicated in the model results (lower sub-panel). **(C)** Honeybee group decisions in modelling a robot-mediated thermal environment with closed-loop control and how this agrees with empirical data (empirical experiments, reported in Stefanec et al., 2017a), and how the modelling results agree with empirical trends. *N* = 14 independent repetitions in each setting. Since the binary choice offered to the bee groups is not *a priori* biassed for one side or the other, we report the number of bees on the majority and minority sides within each repetition; the analysis covers the last 5 min. Three variants of the robot controller, as described in the text, lead to qualitatively different collective decisions by the honeybee group. Specifically, positive feedback linking the local temperature to the local bee density causes strong decision making; negative feedback between bee density and temperature prevents aggregations from building up; the control runs with constant 28°C temperatures throughout are in between and with more variable distributions. The main differences in how strong decision making occurs are reproduced by the model, although once again we see that the variance of distributions from the model, is substantially reduced in comparison with the empirical results.

As Figure 8A demonstrates, the emission of a vibration stimulus leads to an aggregation of bees around the vibrating CASU, compared with the other CASU and compared with the control period. The model predicts this effect in a way very well corresponding to the empirical data. More details are given in Figure 8.

##### Experiment B3: Collective decision making modulated by airflows

In this experiment, two CASUs in a stadium-shaped arena were used. We heated the CASUs for 5 min to different temperature levels: one CASU was heated to TRtarget(t)=36C∘, ∀*t*, further referred to as the global optimum, since young bees prefer to locate at this temperature, as seen already in experiment B1. The other CASU was heated to TLtarget(t)=32C∘, ∀*t*, providing a local optimum for the bees.

We observed groups of *B*_total_ = 15 young (1 day old) honeybees, which were initially released at the centre of the arena. After the bees had stably aggregated at the global optimum after 13–15 min of experimental runtime (*t*_*airflow*_), an airflow stimulus was emitted by the CASU at the global optimum, φ_*R*_(*t*≥*t*_airflow_) = 0.6, until the end of the experiment whose total runtime was 20 min. The control experiments used the same settings, but without turning on the airflow stimulus during the whole runtime. To evaluate the effect of the airflow on the honeybee collective, we counted the bees in the two sides of the arena from video recordings.

As shown in Figure 8B, bees cluster mainly around the warmer CASU before the airflow stimulus is set. After the airflow stimulus has initialised, the initial decision making is reversed, and the bees start to cluster around the cooler CASU. Our model’s predictions compare well with the empirical data. Additional details are given in Figure 8.

##### Experiment B4: The effect of robot-induced feedback on the symmetry breaking in collective decision making

This experiment used a pair of CASUs enclosed by a stadium-shaped arena. In contrast to experiment B1, which showed how bees interact without active robot influence, here, the robots were programmed in a way that they create an additional feedback loop in the system that can enhance or suppress the natural symmetry-breaking capabilities of the bees (Stefanec et al., 2017a). To achieve this, each CASU used its local IR sensors to estimate the local bee density around it and regulated its local temperature in a positive or negative correlation with this estimate (detailed below). The estimated numbers of bees around the left and right CASUs ($B_{L}^{\text{obs}}\left(t\right),B_{R}^{\text{obs}}\left(t\right)$) are modelled assuming that the robots’ IR sensors underestimate the true number of bees (e.g., due to occlusion and blind spots); thus, we model the noise-affected sensor values as

(B-6a) $$B_{R}^{\text{obs}}\left(t\right)=B_{R}\left(t\right)\cdot \left(1-\sigma_{\text{beeCASU}}\cdot X_{R}^{\text{obs}}\left(t\right)\right),$$

(B-6b) $$B_{L}^{\text{obs}}\left(t\right)=B_{L}\left(t\right)\cdot \left(1-\sigma_{\text{beeCASU}}\cdot X_{L}^{\text{obs}}\left(t\right)\right),$$

where σ_*beeCASU*_ is the scaling factor for the observation noise $X_{R}^{\text{obs}}\left(t\right)$, $X_{R}^{\text{obs}}\left(t\right)∼U\left(0,1\right)$, $X_{L}^{\text{obs}}\left(t\right)$$X_{L}^{\text{obs}}\left(t\right)∼U\left(0,1\right)$, assumed to be uniformly distributed. The noise can only lead to underestimation of the number of bees (no false positives in the observation). The CASUs use a gliding average (throughout 30 s), ${\bar{B}}_{R}^{\text{obs}}\left(t\right)\text{and}{\bar{B}}_{L}^{\text{obs}}\left(t\right)$, of the noise-affected sensor values, as can be seen in the following Eqs B7a,b and B8a,b.

*Positive feedback experiments:* A positive feedback means that the CASUs will act to enhance the natural symmetry-breaking behaviour of the bees. To create such a CASU control algorithm, the gliding average number of bees around the ipsilateral CASU was subtracted from the gliding average number of bees around the contralateral CASU to yield the net observed difference. The ipsilateral target temperature had a step increase (decrease) applied when the observed net difference was positive (negative); see Eqs B-7a,b. This led to the effect that the more bees a CASU sensed, the warmer its vicinity got, while at the same time the other CASU became colder (i.e., they exhibited a reciprocal cross-inhibition).

(B-7a) $$T_{R}^{\text{target}}\left(t\right)=\text{min}\left(36.0,\text{ max}\left(28.0,\;T_{R}\left(t\right)+\{\begin{array}{ccc} \Delta_{\text{temp}} & \cdots & \text{if }{\bar{B}}_{R}^{\text{obs}}\left(t\right)>{\bar{B}}_{L}^{\text{obs}}\left(t\right)\\ -\Delta_{\text{temp}} & \cdots & \text{else} \end{array}\right)\right),$$

(B-7b) $$T_{L}^{\text{target}}\left(t\right)=\text{min}\left(36.0,\text{ max}\left(28.0,T_{L}\left(t\right)+\{\begin{array}{ccc} \Delta_{\text{temp}} & \cdots & \text{if }{\bar{B}}_{R}^{\text{obs}}\left(t\right)<{\bar{B}}_{L}^{\text{obs}}\left(t\right)\\ -\Delta_{\text{temp}} & \cdots & \text{else} \end{array}\right)\right).$$

*Negative feedback experiments*: A negative feedback means that the CASUs will act in a way that reduces or even suppresses the natural symmetry breaking behaviour of the bees. To create such a CASU control algorithm, the same observed net difference was calculated but used inversely. Specifically, the ipsilateral target temperature had a step decrease (increase) applied when the observed net difference was positive (negative); see Eqs B-8a,b. Accordingly, the more bees a CASU sensed, the colder its vicinity got, while simultaneously the other CASU became warmer.

(B-8a) $$T_{R}^{\text{target}}\left(t\right)=\text{min}\left(36.0,\text{ max}\left(28.0,T_{R}\left(t\right)+\{\begin{array}{ccc} \Delta_{\text{temp}} & \cdots & \text{if }{\bar{B}}_{R}^{\text{obs}}\left(t\right)<{\bar{B}}_{L}^{\text{obs}}\left(t\right)\\ -\Delta_{\text{temp}} & \cdots & \text{else} \end{array}\right)\right),$$

(B-8b) $$T_{L}^{\text{target}}\left(t\right)=\text{min}\left(36.0,\text{ max}\left(28.0,T_{L}(t)+\{\begin{array}{ccc} \Delta_{\text{temp}} & \cdots & \text{if }{\bar{B}}_{R}^{\text{obs}}\left(t\right)>{\bar{B}}_{L}^{\text{obs}}\left(t\right)\\ -\Delta_{\text{temp}} & \cdots & \text{else} \end{array}\right)\right).$$

*Control experiments*: For comparison, experiments without any reinforcement were conducted; the CASU target temperatures were set to a fixed value of TRtarget(t)=TLtarget(t)=28C∘ on each side, with no influence, neither from bees nor from other CASUs.

All experiments were performed with groups of *B*_total_ = 12 bees each, which were released at the centre of the arena. Each run lasted for 20 min, and we made *N=14* repetitions. In our analysis, we counted the bees on each side of the arena in 30-s intervals from video recordings, which were conducted under red-light conditions, to emulate the darkness of a beehive.

Figure 8C compares a modelled closed loop with empirical data. In both cases, a robot-mediated feedback loop enhanced (positive feedback) or weakened (negative feedback) the natural symmetry breaking of honeybees as compared with the control experiments. Our model’s predictions correspond well to observed empirical data concerning the centrality metric (median); however, the variances within and between model prediction runs are rather small as compared with empirical observations, likely due to the simplicity of the model, having many factors abstracted away from the system. Further details are described in Figure 8.

### Fish and Robot Experimentation

**FIGURE 9:**
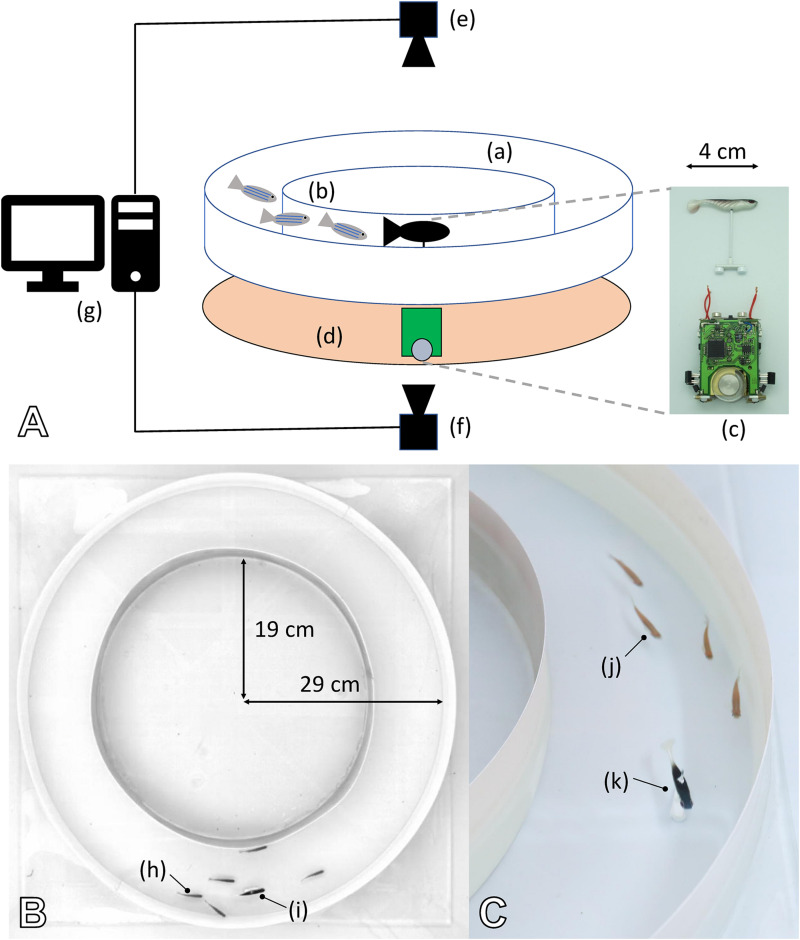
Experimental set-up created to study the interactions in mixed groups composed of fish and one or multiple robots. **(A)** (a) Experimental arena composed of two circular walls forming a circular corridor to condition the behaviour of the agents (see also **B**). (b) Zebrafish moving inside the corridor. (c) The fish robot is composed of a miniature mobile robot (FishBot) and a lure, which is magnetically coupled with the FishBot. (d) Support in which the FishBots are moving, which provides the powering of the system for long-duration experiments. (e) Top camera, which captures the images that are used to determine the position of the agents in real time. (f) Bottom camera, which captures the images to determine the position of the FishBot. (g) Computer running the CATS software for tracking and closed-loop control of the robots in real time. **(B,C)** The arena is composed of two circular walls of 19- and 29-cm radius, which forms a circular corridor of 10-cm width in which the zebrafish (h,j) can move with the robot (i,k). With this configuration, the zebrafish shoal in either the CW or CCW direction, and we can use one or several bio-mimetic robots to blend in with the shoal and influence the swimming direction. **(B)** The top view from the top camera that is used to process the positions of the agents.

To investigate the capability of mobile robots to interact with zebrafish, we performed experiments in which bio-mimetic robots used their motion patterns to exert an influence on the group dynamics of the natural fish. The fish robot consists of two parts: a miniature wheeled robot below the tank that steers a lure residing inside the tank (Figure 9A). The two parts are coupled by magnets, and the partitioning enables continuous power and dry operating conditions for the electro-mechanical devices.

Zebrafish is a social species of fish that exhibit collective behaviours such as shoaling (Spence et al., 2008). The zebrafish was selected as it is a very common model of vertebrates, used in various research fields, in particular in behavioural biology (Norton and Bally-Cuif, 2010). Since visual stimuli are very important in zebrafish interactions, certain aspects of the robot are crucial for the natural fish to interact with the robots and accept them in their decision making. These include the shape and size ratio of the lure, as well as the speed and acceleration of the robot (Bonnet et al., 2018). These robot-generated stimuli were all within the natural ranges of the fish.

Our experiments aimed to verify that a fish robot could influence the group dynamics in two distinct modes: to exert an influence in the swimming direction of the group, (1) where the robot choice decided exogenously (e.g., fixed direction, predetermined pattern, or the experimenter) and (2) in a closed loop where the fish robot direction was chosen to reinforce the current fish group decision.

We selected the fish group size to exhibit some shoaling but also allow for synthetic influence when introducing a small number of robotic agents; the experiments here used a total of 6 agents (6 fish, 3 fish + 3 robots or 5 fish + 1 robot).

The zebrafish used in the studies here was approved by the state ethical board for animal experiments under authorisation number 2778 from the DCVA of Canton de Vaud, Switzerland. As described in Bonnet et al. (2019), we used 100 wild-type, short-fin zebrafish (*Danio rerio* Hamilton 1822) with average length of 4 cm, sourced from Qualipet (Crissier, Switzerland). Each fish could be used in a maximum of one experiment per day, and all fish used were returned to their main tank at the end of the day, meaning that the same individuals could appear in multiple replicates of the studies presented here.

#### The Model of Robots and Fish

The basic principle of the fish and robot model is similar to the concept of the honeybee and robot model. We have a certain number of fish *F*_*total*_, which can swim in the arena ring in either the clockwise direction *F*_*CW*_(*t*) or counter-clockwise direction *F*_*CCW*_(*t*). Initially, they are assumed to be symmetrically split up; thus, *F*_*CW*_(0) = *F*_*CCW*_(0)^1^. Our model, like in the honeybee case, obeys conservation of mass; thus, *F*_*CW*_(*t*) + *F*_*CCW*_(*t*) = *F*_total_.

The fish have a natural behaviour that determines when they switch their locomotion direction, which can either happen as an individual spontaneous event or be triggered by social interaction, within which the fish robot can also participate and exert thus some control over the group of fish. The change between the two groups of fish aligned in each direction is expressed as

(F-1a) $$\begin{array}{c} \frac{dF_{\text{CW}}}{dt}={\text{switch}}_{\text{CW}}^{\text{indiv}}\left(t\right)-{\text{switch}}_{\text{CCW}}^{\text{indiv}}\left(t\right)\\ \;+{\text{switch}}_{\text{CW}}^{\text{social}}\left(t\right)-{\text{switch}}_{\text{CCW}}^{\text{social}}\left(t\right), \end{array}$$

(F-1b) $$\begin{array}{c} \frac{dF_{\text{CCW}}}{dt}={\text{switch}}_{\text{CCW}}^{\text{indiv}}\left(t\right)-{\text{switch}}_{\text{CW}}^{\text{indiv}}\left(t\right)\\ \;+{\text{switch}}_{\text{CCW}}^{\text{social}}\left(t\right)-{\text{switch}}_{\text{CW}}^{\text{social}}\left(t\right), \end{array}$$

where ${\text{switch}}_{\text{CW}}^{\text{indiv}}\left(t\right)$ represents the number of fish individually switching from the CCW to CW direction, and ${\text{switch}}_{\text{CCW}}^{\text{indiv}}\left(t\right)$ models the individual process of switching into the opposite direction. The variables ${\text{switch}}_{\text{CW}}^{\text{social}}\left(t\right)$ express fish that switch to the CW direction triggered by a social interaction, while ${\text{switch}}_{\text{CCW}}^{\text{social}}\left(t\right)$ expresses the opposite socially induced switching of direction.

*Individual direction switching*: On the one hand, the direction-changing process can happen spontaneously without any triggering event. We assume that this happens with a certain rate α_fish_ whenever a fish is alone in the tank and, thus, has no other fish (or fish robot) in sight that can socially influence it. The fraction of the fish population that is predicted to be alone is modelled as

(F-2a) $$p_{\text{alone}}=1-p_{\text{group}},$$

(F-2b) $$p_{\text{group}}=\text{min}\left(1.0,\frac{F_{\text{total}}\cdot A_{\text{sight}}}{A_{\text{arena}}}\right),$$

where *A*_arena_ represents the area of the ring-shaped arena and *A*_sight_ represents the area of the cone of sight of a single fish in this arena shape. Geometrical considerations show that the field of perception of a fish covers roughly between $\frac{1}{3}$ (if the fish is close to the outer arena wall) and $\frac{1}{7}$ (if the fish is close to the inner wall) of *A*_arena_; thus, we assume an average coverage of approximately $\frac{1}{5}$ of this area for *A*_sight_. We further assume, in our mean-field model, that at a given number of fish in the arena, no fish will ever be alone. With a given probability of α_fish_, a fish that is alone will switch to swimming in the opposite direction, as is expressed by

(F-3a) $${\text{switch}}_{\text{CW}}^{\text{indiv}}\left(t\right)=\alpha_{\text{fish}}\cdot p_{\text{alone}}\cdot F_{\text{CCW}}\left(t\right),$$

(F-3b) $${\text{switch}}_{\text{CCW}}^{\text{indiv}}\left(t\right)=\alpha_{\text{fish}}\cdot p_{\text{alone}}\cdot F_{\text{CW}}\left(t\right).$$

*Socially induced direction switching*: On the other hand, fish can also switch to the opposite direction because they see other fish and want to align with their motion direction. This is modelled, similar to the previous honeybee model, with a mass-action-law-like equation, modulated by a coefficient β_fish_, which determines the strength of this socially induced direction switching (Eqs F-6a,b).

We assume that each fish has an imperfect perception of the direction of the other fish it sees; thus, it only has an erroneous estimation of the number of fish swimming aligned with it or in the opposite direction. For a fish that is currently swimming CW, the estimated number of other fish also swimming CW is modelled by $F_{\text{CW}}^{\text{obsCW}}\left(t\right)$, and the estimation for swimming CCW is modelled by $F_{\text{CCW}}^{\text{obsCW}}\left(t\right)$. These variables are computed as

(F-4a) $$F_{\text{CW}}^{\text{obsCW}}\left(t\right)=F_{\text{CW}}\left(t\right)+E_{\text{CW}}^{\text{obsCW}}\left(t\right)-E_{\text{CCW}}^{\text{obsCW}}\left(t\right),$$

(F-4b) $$F_{\text{CCW}}^{\text{obsCW}}\left(t\right)=F_{\text{CCW}}\left(t\right)-E_{\text{CW}}^{\text{obsCW}}\left(t\right)+E_{\text{CCW}}^{\text{obsCW}}\left(t\right),$$

where $E_{\text{CCW}}^{\text{obsCW}}\left(t\right)$ is the number of fish swimming in the same direction (CW) but erroneously perceived by the CW swimming fish as being swimming in the CCW direction. $E_{\text{CW}}^{\text{obsCW}}\left(t\right)$ is the number of fish swimming in the opposite direction (CCW) but erroneously perceived by the CW-swimming fish as being aligned with them (CW). These errors in the fish observation are modelled as

(F-5a) $$E_{\text{CCW}}^{\text{obsCW}}\left(t\right)=\sigma_{\text{fish}}\cdot \left(F_{\text{CW}}\left(t\right)-1\right)\cdot X_{\text{CW}}\left(t\right),$$

(F-5b) $$E_{\text{CW}}^{\text{obsCW}}\left(t\right)=\sigma_{\text{fish}}\cdot F_{\text{CCW}}\left(t\right)\cdot X_{\text{CCW}}\left(t\right),$$

where *X*_CW_(*t*)∼*U*(0,1) and *X*_CCW_(*t*)∼*U*(0,1) are the noise parameters and σ_fish_ is a scaling coefficient for the perception error. A similar computation holds for the variables $F_{\text{CW}}^{\text{obsCCW}}\left(t\right)$ and $F_{\text{CCW}}^{\text{obsCCW}}\left(t\right)$ as the erroneous observations made by the fish swimming CCW concerning the other fish they see, as

(F-4c) $$F_{\text{CW}}^{\text{obsCCW}}\left(t\right)=F_{\text{CW}}\left(t\right)+E_{\text{CW}}^{\text{obsCCW}}\left(t\right)-E_{\text{CCW}}^{\text{obsCCW}}\left(t\right),$$

(F-4d) $$F_{\text{CCW}}^{\text{obsCCW}}\left(t\right)=F_{\text{CCW}}\left(t\right)-E_{\text{CW}}^{\text{obsCCW}}\left(t\right)+E_{\text{CCW}}^{\text{obsCCW}}\left(t\right),$$

(F-5c) $$E_{\text{CCW}}^{\text{obsCCW}}\left(t\right)=\sigma_{\text{fish}}\cdot F_{\text{CW}}\left(t\right)\cdot X_{\text{CW}}\left(t\right),$$

(F-5d) $$E_{\text{CW}}^{\text{obsCCW}}\left(t\right)=\sigma_{\text{fish}}\cdot \left(F_{\text{CCW}}\left(t\right)-1\right)\cdot X_{\text{CCW}}\left(t\right)$$

where the noise variables are modelled as *X*_CW_(*t*)∼*U*(0,1) and *X*_CCW_(*t*)∼*U*(0,1).

For the fish switching direction due to social effects, our model assumes the following social alignment behaviour for each focal fish: If a large proportion of others swim aligned with it, the tendency for switching is low. If a large proportion is swimming in the opposite direction, the fish tends to switch its own direction. This behaviour is again modelled following the mass action law, as was also the case in the honeybee model. The number of fish in CCW switching to CW depends on the number of fish in CCW and a function of their erroneous observations they make concerning other fish they meet ($F_{\text{CW}}^{\text{obsCCW}}\left(t\right)$ and $F_{\text{CCW}}^{\text{obsCCW}}\left(t\right)$). Thus, the social switching functions are directly correlated with their estimated number for CW swimming fish, $F_{\text{CW}}^{\text{obsCCW}}\left(t\right)$, and inversely correlated with their estimated number for CCW swimming fish, $F_{\text{CCW}}^{\text{obsCCW}}\left(t\right)+1$. The + 1 term in the equation refers to each focal fish. The following equations show the model for switching to CW and CCW, respectively:

(F-6a) $$\begin{array}{c} {\text{switch}}_{\text{CW}}^{\text{social}}\left(t\right)=\beta_{\text{fish}}\cdot p_{\text{group}}\cdot F_{\text{CCW}}\left(t\right)\cdot \\ \;\;\frac{F_{\text{CW}}^{\text{obsCCW}}\left(t\right)}{{\text{F}}_{\text{CCW}}^{\text{obsCCW}}\left(t\right)+1}, \end{array}$$

(F-6b) $$\begin{array}{c} {\text{switch}}_{\text{CCW}}^{\text{social}}\left(t\right)=\beta_{\text{fish}}\cdot p_{\text{group}}\cdot F_{\text{CW}}\left(t\right)\cdot \\ \;\;\frac{F_{\text{CCW}}^{\text{obsCW}}\left(t\right)}{F_{\text{CW}}^{\text{obsCW}}\left(t\right)+1}. \end{array}$$

In our experiments, we also introduced one or more fish robots that mimicked real fish. We assume that the living fish perceived the fish robot as conspecific, but perhaps not to the full extent. Thus, we define a coefficient γ_fish_ ∈ [0,1]expressing how often (in all instances of encounters) the fish robot was interpreted by the living fish as a conspecific. This presence of a robotic fish surrogate needs to be considered in the model, requiring a reformulation of Eqs F-2a,b into

(F-2c) $$p_{\text{alone}}=1-p_{\text{group}},$$

(F-2d) $$p_{\text{group}}=\text{min}\left(1.0,\frac{\left(F_{\text{total}}+\gamma_{\text{fish}}\right)\cdot A_{\text{sight}}}{A_{\text{arena}}}\right)$$

which will have a small effect on the spontaneous direction switching behaviour expressed in Eqs F-3a,b and also on the socially induced direction switching behaviour, as expressed by Eqs F-4a,b.

Further beyond the mere presence of another fish-like agent, its direction can have profound effects on the socially induced direction switching behaviour of the fish. Thus, we express the fish robot as a variable *R*_CW_(*t*) ∈ [0,1] expressing how much of the time budget the fish robots swam in the CW direction. Consequently, *R*_CCW_(*t*) = 1-*R*_CW_(*t*) and *R*_CW_(*t*) + *R*_CCW_(*t*) = 1. This requires the alteration of Eqs F-4a–d to also consider the social effect of the fish robot, as

(F-4e) $$\begin{array}{c} F_{\text{CW}}^{\text{obsCW}}\left(t\right)=F_{\text{CW}}\left(t\right)+\gamma_{\text{fish}}\cdot R_{\text{CW}}\left(t\right)\\ \;+E_{\text{CW}}^{\text{obsCW}}\left(t\right)-E_{\text{CCW}}^{\text{obsCW}}\left(t\right), \end{array}$$

(F-4f) $$\begin{array}{c} F_{\text{CCW}}^{\text{obsCW}}\left(t\right)=F_{\text{CCW}}\left(t\right)+\gamma_{\text{fish}}\cdot R_{\text{CCW}}\left(t\right)\\ \;-E_{\text{CW}}^{\text{obsCW}}\left(t\right)+E_{\text{CCW}}^{\text{obsCW}}\left(t\right), \end{array}$$

(F-4g) $$\begin{array}{c} F_{\text{CW}}^{\text{obsCCW}}\left(t\right)=F_{\text{CW}}\left(t\right)+\gamma_{\text{fish}}\cdot R_{\text{CW}}\left(t\right)\\ \;+E_{\text{CW}}^{\text{obsCCW}}\left(t\right)-E_{\text{CCW}}^{\text{obsCCW}}\left(t\right), \end{array}$$

(F-4h) $$\begin{array}{c} F_{\text{CCW}}^{\text{obsCCW}}\left(t\right)=F_{\text{CCW}}\left(t\right)+\gamma_{\text{fish}}\cdot R_{\text{CCW}}\left(t\right)\\ \;-E_{\text{CW}}^{\text{obsCCW}}\left(t\right)+E_{\text{CCW}}^{\text{obsCCW}}\left(t\right). \end{array}$$

In addition, the erroneous perception of fish, as described in Eqs F-5a–d, has to be adapted to model also the effect of the fish robot, which can also be erroneously perceived, as

(F-5e) $$\begin{array}{c} E_{\text{CCW}}^{\text{obsCW}}\left(t\right)=\sigma_{\text{fish}}\cdot (F_{\text{CW}}\left(t\right)+\gamma_{\text{fish}}\cdot \\ \;\;R_{\text{CW}}\left(t\right)-1)\cdot X{}_{\text{CW}}{}^{\text{obsCW}}(t), \end{array}$$

(F-5f) $$\begin{array}{c} E_{\text{CW}}^{\text{obsCW}}\left(t\right)=\sigma_{\text{fish}}\cdot (F_{\text{CCW}}\left(t\right)+\gamma_{\text{fish}}\cdot \\ \;\;R_{\text{CCW}}\left(t\right))\cdot X{}_{\text{CCW}}{}^{\text{obsCW}}(t), \end{array}$$

(F-5g) $$\begin{array}{c} E_{\text{CCW}}^{\text{obsCCW}}\left(t\right)=\sigma_{\text{fish}}\cdot (F_{\text{CW}}\left(t\right)+\gamma_{\text{fish}}\cdot \\ \;\;R_{\text{CW}}\left(t\right))\cdot X{}_{\text{CW}}{}^{\text{obsCCW}}(t), \end{array}$$

(F-5h) $$\begin{array}{c} E_{\text{CW}}^{\text{obsCCW}}\left(t\right)=\sigma_{\text{fish}}\cdot (F_{\text{CCW}}\left(t\right)+\gamma_{\text{fish}}\cdot \\ \;R_{\text{CCW}}\left(t\right)-1)\cdot X{}_{\text{CCW}}{}^{\text{obsCCW}}(t), \end{array}$$

where $X_{\text{CW}}^{\text{obsCW}}\left(t\right),X_{\text{CCW}}^{\text{obsCW}}\left(t\right),X_{\text{CW}}^{\text{obsCCW}}\left(t\right),X_{\text{CCW}}^{\text{obsCCW}}\left(t\right)∼U\left(0,1\right)$.

Ultimately, these components all affect the social behaviour of the fish, thus requiring the adaptation of Eqs F-6a,b to

(F-6c) $$\begin{array}{c} {\text{switch}}_{\text{CW}}^{\text{social}}\left(t\right)=\beta_{\text{fish}}\cdot p_{\text{group}}\cdot \left(F_{\text{CCW}}\left(t\right)+\gamma_{\text{fish}}\cdot R_{\text{CCW}}\left(t\right)\right)\cdot \\ \;\;\frac{F_{\text{CW}}^{\text{obsCCW}}\left(t\right)}{F_{\text{CCW}}^{\text{obsCCW}}\left(t\right)+1}, \end{array}$$

(F-6d) $$\begin{array}{c} {\text{switch}}_{\text{CCW}}^{\text{social}}\left(t\right)=\beta_{\text{fish}}\cdot p_{\text{group}}\cdot \left(F_{\text{CW}}\left(t\right)+\gamma_{\text{fish}}\cdot R_{\text{CW}}\left(t\right)\right)\cdot \\ \;\;\frac{F_{\text{CCW}}^{\text{obsCW}}\left(t\right)}{F_{\text{CW}}^{\text{obsCW}}\left(t\right)+1}. \end{array}$$

In the following, we describe three distinct experiments, in which the fish robots were performing different types of behaviour. In the first two experiments, the robots acted independently, without being affected by the fish, allowing us to study the fish reaction to this external visual stimulus. In the third experiment, the fish robot was trying to socially integrate into the fish group by aligning with the fish, thus closing the behavioural feedback loop between the fish and the fish robot. The default parameters for the model are defined in Figure 2B.

#### Experiments With Robots and Fish

Inside a 100 × 100 × 25 cm aquarium covered with white Teflon sheets, the experimental set-up used a circular corridor for the fish and robot-controlled lure to move in Figures 9B,C. The water was filled to a level of 6 cm and maintained at 26°C. The arena was lit by three 110-W fluorescent lamps and continuously observed by an overhead camera at 15 Hz. The video stream fed an online blob detector that continuously determined the position of each fish and robot, thereby providing the sensory information used to determine the robot motion (Bonnet et al., 2017a). *Post hoc* analysis of the videos used idTracker (Pérez-Escudero et al., 2014) and provided individual tracking as well as lower-error position information. For a detailed description of the set-up and robot controller, please refer to Bonnet et al. (2018).

##### Experiment F1: Fish group behaviour in pure groups and mixed groups with constant robotic influence

**FIGURE 10:**
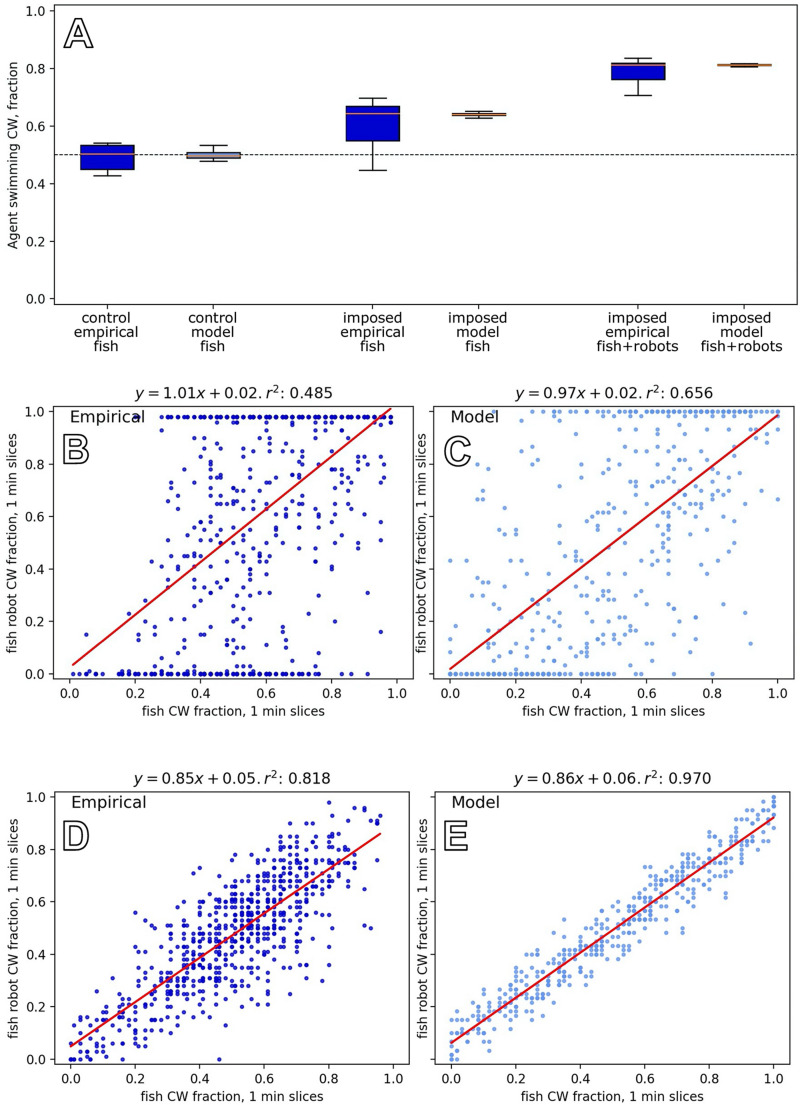
Results of model and empirical data from experiments with robots and fish groups, experiments F1–F3. **(A)** Comparing group-level direction choices between six fish (left) and a mixed group of three fish with three robots that constantly swam in the same direction (the right shows the whole group, and the middle shows data for the three fish in the context of robots). Trends in the empirical data, from *N=8* repetitions (Bonnet et al., 2018) are reflected in the model output. **(B)** Experiments with five fish and one fish robot that had an exogenously defined motion, switching direction in 1.4% of the time steps, reveals a correlation between the swimming direction of the fish group and the robot (empirical data from Bonnet et al., 2019 with *N=24* repetitions). **(D)** Experiments with five fish and one fish robot that acted to reinforce the swimming direction of the fish group (empirical data from Bonnet et al., 2019, with *N=22* repetitions). The relationship between the fish robot direction and fish group decision is tighter in this closed-loop setting than in the open-loop setting above. **(C,E)** Equivalent output from our model for experiments F2 and F3, showing the same trends as the empirical results.

To investigate the natural grouping behaviour of the fish without robotic influence, we tested groups of six zebrafish in the arena (Bonnet et al., 2018). As a first comparison, we tested mixed groups of three fish and three fish robots, where the fish robots swam in the same direction for each of the *N* = 8 experiments that lasted for 30 min. Figure 10A shows empirical results and how the model reproduces the key dynamics in both cases. It shows that fish were influenced to swim with the robots when the robots swam constantly in one direction, in contrast to the unbiassed swimming direction with pure fish groups. The empirical result is well captured by our model.

##### Experiment F2: Mixed fish and robot groups, with independent fish robot motion

In this experiment, we constructed mixed groups of five fish and one robot (Bonnet et al., 2019). In contrast to experiment F1, the robot exhibited various direction changes, which were specified independently from the swimming direction of the fish group (changing direction with a frequency of 0.014 ± 0.006 per time step). The experiments lasted 30 min, and we conducted *N=24* repetitions. To govern the fish robot direction in the model, we used a simple two-state machine that switched direction with probability 0.014 in each time step. Figure 10B shows the relationship between the fish group choice and the robot swimming direction, which is positively correlated with a wide distribution. The model reproduces these dynamics (Figure 10C).

##### Experiment F3: Fish Robot in “social integration” mode, a closed-loop setting with the fish group behaviour

In a manner similar to that of experiment B4, the robots in this experiment form a closed loop with the animal behaviour, aiming to reinforce the current decision of the animal group. We used five fish and one robot that swam in the majority direction of the fish group. We conducted *N=22* repetitions of 30-min long experiments. The fish are modelled as per the previous experiments, responding to their environmental cues including the robot. However, here, the model must also consider how the robot responds to the fish locomotion, as elaborated below.

To decide on the swimming direction of the robotic fish, the robot controller computes the proportion of the fish observed in each direction for 15 frames in every second. It then averages these values and decides on its future direction based on this calculated time budget. Since we use a time step of Δ*t* = 1 s in our model, the modelled controller computes a single proportion in every second.

The robot’s decision is modelled as

(F-7a) $$R_{\text{CW}}\left(t\right)=\left\{ \begin{array}{c} 1\\ -1 \end{array}\right.\begin{array}{c} \cdots \;\text{if}F_{\text{CW}}^{\mathrm{obsR}}\left(t\right)>0.5\\ \cdots \;\mathrm{if}F_{\text{CW}}^{\mathrm{obsR}}\left(t\right)<0.5 \end{array},$$

(F-7b) $$R_{\text{CCW}}\left(t\right)=1-R_{\text{CW}}\left(t\right),$$

where $F_{\text{CW}}^{\text{obsR}}\left(t\right)$ and $F_{\text{CCW}}^{\text{obsR}}\left(t\right)$ are the gliding averages in the CW and CCW directions correspondingly. If there is a tie between the two possible directions, a random direction is chosen by the robotic fish CASU.

In order to compute the proportions to make the gliding averages, the number of fish in each direction observed by the detection software is divided by the total number of fish. The online fish detection software (CATS, Bonnet et al., 2017a) that informs the controller of the robotic fish is imperfect in detecting directions. The erroneous observed proportions of the number of fish are modelled as the true number of fish in each direction [*F*_CW_(*t*), *F*_CCW_(*t*)], plus the error [$R_{\text{CW}}^{\text{error}}\left(t\right)$,$R_{\text{CCW}}^{\text{error}}\left(t\right)$], divided by the total number of fish, in order to normalise for the given fish size.

(F-8a) $$F_{\text{CW}}^{\text{obsR}}\left(t\right)=\frac{F_{\text{CW}}\left(t\right)+R_{\text{CW}}^{\text{error}}\left(t\right)}{F_{\text{total}}},$$

(F-8b) $$F_{\text{CCW}}^{\text{obsR}}\left(t\right)=\frac{F_{\text{CCW}}\left(t\right)+R_{\text{CCW}}^{\text{error}}\left(t\right)}{F_{\text{total}}},$$

where $R_{\text{CW}}^{\text{error}}\left(t\right)$ is the error in the observed number of fish swimming in the CW direction and $R_{\text{CCW}}^{\text{error}}\left(t\right)$ is the error in the observed number of fish in the CCW direction made by the software that observes the real fish to drive the robot. This error is modelled as

(F-9a) $$\begin{array}{c} R_{\text{CW}}^{\text{error}}\left(t\right)=\sigma_{\text{fishRobot}}\cdot X_{\text{CCW}}\left(t\right)\cdot F_{\text{CCW}}\left(t\right)\\ \;-\sigma_{\text{fishRobot}}x\cdot X_{\text{CW}}\left(t\right)\cdot F_{\text{CW}}\left(t\right), \end{array}$$

(F-9b) $$R_{\text{CCW}}^{\text{error}}\left(t\right)=-R_{\text{CW}}^{\text{error}}\left(t\right),$$

where the random noise variables were modelled as *X*_CW_(*t*)∼*U*(0,1) and *X*_CCW_(*t*)∼*U*(0,1) with uniform distribution, and σ_fishRobot_ is the scaling factor for the observation noise. In this model, the number of fish swimming in the CW direction but mistakenly counted as the CCW direction is modelled as σ_fishRobot_⋅*X*_CW_(*t*)⋅*F*_CW_(*t*); and the number of fish swimming in CCW but mistakenly counted as the CW direction is σ_fishRobot_⋅*X*_CCW_(*t*)⋅*F*_CCW_(*t*).

Figures 10D,E show the dynamics of this closed-loop system, exhibiting a high correlation between the robot and fish group choices in this closed-loop system (cf. especially Figures 10B,C).

### Plant and Robot Experimentation

We focus here on the capability of robots to interact with growing plant shoots (here, the common bean, *Phaseolus vulgaris* L.). CASU nodes (i) detected the presence of plants and (ii) altered the local environment by providing light stimuli. The young bean shoots bend and favour their growth towards the strongest incident light in a process called phototropism (see e.g., Christie and Murphy, 2013). This allows for feedback loops between the CASUs’ and plants’ behaviours to be constructed.

Two general approaches were followed, different in scale (in space and time) and precision. (1) A system consisting of a single board computer with a camera and control over two light sources together with a single freshly sprouted bean plant was used to guide the growing shoots to multiple targets in space using image detection and machine learning (detailed in Hofstadler et al., 2017). In these experiments, it typically took the bean shoot 2–3 days to grow out of the space monitored by the camera, corresponding to ∼50 cm of bean shoot. We showcase the model laid out below by simulating such a system. (2) A decentralised group of plant CASUs was attached to a scaffold that allowed the plants to climb vertically (Figure 5E). These CASUs can detect plants that are still below them via IR-distance sensors, and they can attract these plant shoots to grow towards them with a set of strong LEDs. In this setting, many individual plants climb up the scaffold across multiple layers of robots during the course of ∼2 months. A detailed account is given in Wahby et al. (2018).

#### The Model of Robots and Plants

Plant shoots grow upwards by producing new cells at the tip (Wang et al., 2018). Below the tip, cells elongate and mature. This upper zone of a growing stem (roughly the top 10 cm in beans) is flexible and rotates around the central stem-axis autonomously, a process called “circumnutation” (Stolarz, 2009; Mugnai et al., 2015). The plant co-opts and overrides this basic behaviour to quickly react to environmental cues. If, for example, light suddenly comes from a different angle, the flexible zone will quickly bend towards it (by elongating cells on the far side). On a whole-plant level, multiple growing tips generated via branching (Barbier et al., 2019) strongly influence each other’s growth capacity (see e.g., Bennett et al., 2016; Zahadat and Hofstadler, 2019). But here the focus lies solely on the growth and motion of a single plant tip under the influence of light stimuli.

The presented model describes the dynamics of the flexible part of a single bean stem *P*^flex^(*t*) growing through the system (the biomass of the mature, stiff stem is not considered). Like in the honeybee model shown before, space is divided in the left and right regions that may contain flexible plant mass. In the following, the subscripts “*L*” and “*R*” refer to the left and right sides, respectively; e.g., $P_{L}^{\text{flex}}\left(t\right)$indicates the flexible plant mass on the left side at time *t*. In contrast to the bee model, space here has an additional implicit vertical component: Flexible plant mass enters the system via growth through a central stock *P*^stem^(*t*), from where it is divided between $P_{L}^{\text{flex}}\left(t\right)$ and $P_{R}^{\text{flex}}\left(t\right)$. From there on, flexible plant mass may switch sides or leave the system. Switching sides in the model corresponds to bending of the plant stem. An equal distribution of mass between the left and right means that the plant has grown a perfectly upright stem.

Combined actuator sensor units above each lateral compartment detect plants below themselves and adjust light emissions accordingly, thereby influencing the lateral movements of the plant tips. These CASUs are not explicitly modelled; instead, the variable Λ(*t*) models the ratio between the two light intensities. The outgrowth terms correspond to the amounts of plant biomass that grows out of our model’s reference frame over time. Consequently, the plant biomass changes in the three modelled state variables are given by balancing the flows between them in a system of three difference equations^2^, as is expressed by

(P-1a) $$\frac{\text{Δ}P^{\text{stem}}}{\text{Δ}t}=\text{ingrowth}\left(t\right)-{\text{growth}}_{R}\left(t\right)-{\text{growth}}_{L}\left(t\right),$$

(P-1b) $$\begin{array}{ccc} \frac{\Delta P_{R}^{\text{flex}}}{\Delta t}={\mathrm{growth}}_{R}\left(t\right)+{\text{switch}}_{R}^{\text{indiv}}\left(t\right)+{\text{switch}}_{R}^{\text{social}}\left(t\right) & & \\ \;-{\text{switch}}_{L}^{\text{indiv}}\left(t\right)-{\text{switch}}_{L}^{\text{social}}\left(t\right)-{\text{outgrowth}}_{R}\left(t\right), & & \end{array}$$

(P-1c) $$\begin{array}{ccc} \frac{\Delta P_{L}^{\text{flex}}}{\Delta t}={\text{growth}}_{L}\left(t\right)+{\text{switch}}_{L}^{\text{indiv}}\left(t\right)+{\text{switch}}_{L}^{\text{social}}\left(t\right) & & \\ \;-{\text{switch}}_{R}^{\text{indiv}}\left(t\right)-{\text{switch}}_{R}^{\text{social}}\left(t\right)-{\text{outgrowth}}_{L}\left(t\right). & & \end{array}$$

The individual flows of Eqs P-1a–c are detailed in the following equations. Plant mass enters the system exclusively via a constant growth rate adding to the system variable *P*^stem^(*t*):

(P-2) $$\text{ingrowth}\left(t\right)=\rho_{\text{in}},$$

where ρ_in_ is the growth rate determining the influx into the system. Next, the already-existing plant biomass in *P*^stem^(*t*) grows further upwards and is split into additions to the system variables that model plant biomass on the left and right sides:

(P-3a) $${\text{growth}}_{R}\left(t\right)=P^{\text{stem}}\left(t\right)/2,$$

(P-3b) $${\text{growth}}_{L}\left(t\right)=P^{\text{stem}}\left(t\right)/2.$$

Plant mass can switch between these two sides via two basic mechanisms: with and without interactions with plant mass on the contralateral side. The individual phototropic movement towards the light is modelled as

(P-4a) $${\text{switch}}_{R}^{\text{indiv}}\left(t\right)=\alpha_{\text{plant}}\cdot X_{R}^{\text{indiv}}\left(t\right)\cdot P_{L}^{\text{flex}}\left(t\right)\cdot \Lambda \left(t\right)$$

and

(P-4b) $$\;{\text{switch}}_{L}^{\text{indiv}}\left(t\right)=\alpha_{\text{plant}}\cdot X_{L}^{\text{indiv}}\left(t\right)\cdot P_{R}^{\text{flex}}\left(t\right)\cdot \left(1-\Lambda \left(t\right)\right),$$

where α_plant_ is a constant parameter controlling the rate (limited by the bean kinetics of circumnutation and phototropism) and two independent, normally distributed noise functions *X*^indiv^(*t*)∼*N*(μ = 1,σ = σ_plant_) with the deviation σ_plant_ ∈ [0,1]. The variable Λ(*t*) ∈ [0,1] models the ratio between the light intensities on the left and right sides, with the value 0.0 corresponding to all light on the left side. More specifically, the definition of Λ(*t*)depends on the capabilities of the used CASUs and the algorithm running on them (see Eqs P-7–9).

Several studies and models (see e.g., Mugnai et al., 2015) attribute the observable circumnutation to the fact that within the growing shoot, cells on opposing sides interact via physical (mechanical) forces. Cells on one side of the elongation zone sometimes grow stronger than those on the opposing side. This asymmetrical growth bends the tip towards the opposing side. However, bending is limited to some extent by the mechanical integrity of the plant: It is expected to be easier for the plant to go from a relaxed (balanced) state to a bent state than to bend even more when already bent. In consequence, we model circumnutation as the social part (which involves interactions of biomass from both sides) of the flows between the sides as

(P-5a) $${\text{switch}}_{R}^{\text{social}}\left(t\right)=\beta_{\text{plant}}\cdot X_{R}^{\text{social}}\left(t\right)\cdot P_{L}^{\text{flex}}\left(t\right)\cdot P_{R}^{\text{flex}}\left(t\right),$$

(P-5b) $${\text{switch}}_{L}^{\text{social}}\left(t\right)=\beta_{\text{plant}}\cdot X_{L}^{\text{social}}\left(t\right)\cdot P_{L}^{\text{flex}}\left(t\right)\cdot P_{R}^{\text{flex}}\left(t\right).$$

Circumnutation is expressed by a normally distributed noise term *X*^social^(*t*)∼*N*(μ = 1,σ = σ_plant_), which scales a mass-action-law term [$P_{L}^{\text{flex}}\left(t\right)\cdot P_{R}^{\text{flex}}\left(t\right)$] to consider the interaction between groups of cells on opposing sides of the plant. This scales the noise amplitude in a way that more change is assumed to arise under balanced conditions and less in already unbalanced configurations. The constant β_plant_ scales this process in proportion to the light-following process, which is weighted by the coefficient α_plant_ (in Eqs P-4a,b). Finally, plant biomass leaves the system by growing out at the top on each side, which is modelled as

(P-6a) $${\text{outgrowth}}_{R}\left(t\right)=\rho_{\text{out}}\cdot P_{R}^{\text{flex}}\left(t\right),$$

(P-6b) $${\text{outgrowth}}_{L}\left(t\right)=\rho_{\text{out}}\cdot P_{L}^{\text{flex}}\left(t\right),$$

with ρ_out_ expressing a constant growth rate coefficient.

The light ratio variable Λ(*t*) ∈ [0,1] models the combined light output of the two robots in a single dimensionless variable that states where light is focused on the horizontal axis of the system. Physical quantities of light are not explicitly modelled: When both robots output the same amount of light (even none), Λ(*t*) = 0.5. Values smaller (larger) than 0.5 model indicate shifts to the left (right). The function generating this value defines the CASU’s capabilities and how they are employed to enable feedback loops in the system.

We define a plant inhomogeneity metric Υ(*t*)to express the imbalance between plant biomass on both sides

(P-7) $$\Upsilon \left(t\right)=0.5\cdot \left(\frac{P_{R}^{\text{flex}}\left(t\right)-P_{L}^{\text{flex}}\left(t\right)}{P_{R}^{\text{flex}}\left(t\right)+P_{L}^{\text{flex}}\left(t\right)+1}+1\right).$$

This inhomogeneity has similar properties as the light ratio Λ(*t*), i.e., Υ(*t*) ∈ (0,1), with 0.5 corresponding to an equal distribution of plant mass between the two sides. The division term computes the relative difference between plants on both sides. However, because of the “+1” in the denominator, the extreme values 0.0 and 1.0 will never be produced, hence the open interval. Very small amounts of total plant mass in the system will produce values close to the centre, analogous to freshly germinated shoots, which are physically unable to move away far from the centre due to their short stem. Increasing plant mass allows for a greater reach of the tip.

We can also interpret the metric Υ(*t*) as a result of the combined plant detection of the two CASUs, allowing us to model simple CASU behaviours that impose positive or negative feedback loops onto the biohybrid system. For example, to model CASUs that emit more light when they detect more plants, a positive feedback function for the light ratio Λ^posFB^(*t*) can be defined:

(P-8) $$\Lambda^{\text{posFB}}\left(t\right)=\Upsilon \left(t\right)+X^{\text{detect}}\left(t\right),$$

with a normally distributed noise function *X*^detect^(*t*)∼*N*(μ = 0,σ = σ_plantCASU_) that accounts for imperfect plant detection by the CASUs. Systems with a light ratio computed this way will only fluctuate shortly (due to the random noise in plant mass movements and plant detection), before concentrating all plant mass on one side. Similarly, the negative feedback function Λ^negFB^(*t*) can be modelled by simply mirroring the plant ratio Υ(*t*):

(P-9) $$\Lambda^{\text{negFB}}\left(t\right)=1-\Upsilon \left(t\right)+X^{\text{detect}}\left(t\right),$$

Here, detected plant mass decreases the light output of a robot. This leads to systems where plant mass is equally distributed between both sides in the long run, with deviations from a perfectly adequate light ratio only due to the detection noise *X*^detect^(*t*). Noise in plant motion (*X*^social^(*t*) and *X*^indiv^(*t*)) causes additional fluctuations around an equal distribution of plant mass.

A value of Λ(*t*)other than 0.0, 0.5, or 1.0 does not necessarily mean that the CASUs need to be able to modify the intensity of the light they emit but can also be understood as the ratio between the relative times that each CASU was switched on within the time window corresponding to a single time step in our model. Conversely, binary functions (that return either zero or one) for a given time step can be defined just as well. Such a binary function is utilised in the experiment described in the next section (Eq. P-10).

#### Experiments With Robots and Plants

**FIGURE 11:**
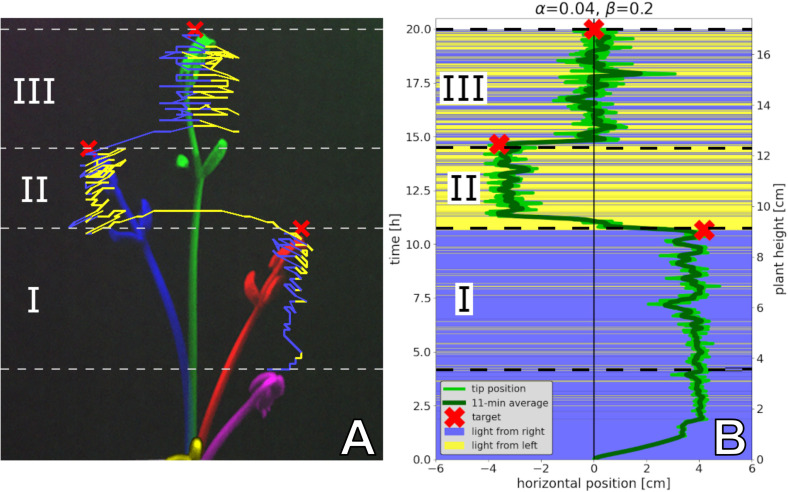
Plant experiment and simulation of binary light control guiding a plant tip to hit three targets (shown with red crosses) during growth. **(A)** The image is compiled from five different time steps of an experiment reported in Hofstadler et al. (2017). For each time step shown, the bean is mapped to a different colour; the tip’s trajectory through the 2D projection plane of the images throughout the experiment is overlaid (in yellow when the light comes from the left, and blue otherwise). The emerging seedling is shown in yellow (bottom centre). In magenta, we see the bean when the plant tip was first detected. This marks the beginning of phase I, where light mainly comes from the right side in order to keep the bean below the first target (at a height of 9 and 4 cm to the right) until it is reached (red bean). In phase II, the bean is guided to the second target (height = 12 cm, 3.5 cm to the left) on the left. Note the fast reaction (∼15 min) indicated by the yellow curve of the trajectory from the first target towards the left side, when the light regime changes. Thereafter, phase II is again characterised by the typical oscillations (due to circumnutation) below the target until it is reached (blue bean). In phase III, the target is located centrally (height = 17 cm), leading to frequent light switching and larger horizontal movements of the tip. The bean drawn in green has reached this final target. **(B)** A simulation run of the plant model (with parameters according to Figure 2C). The vertical axis represents time (at 1-min resolution), instead of the actual position projected onto the image plane. The targets have been placed according to the simplification of a linear conversion of time into height (ignoring geometrical constraints and assuming a constant growth rate). This implies that no downward motion of the tip is to be expected, since time progresses linearly in our model. Our model aims to describe a plant tip’s behaviour from germination onwards, while in the experiments with real plants, tip detection only kicked in at a height of ∼3.5 cm. There is thus no basis for a comparison for these early time steps. Furthermore, the model’s parameters are not tuned to accurately represent this very early phase of growth. During phases I–III, behaviours very similar (qualitatively) to those of the real plants can be observed. Keeping the tip below targets far from the central axis requires light from the according direction most of the time. The final and central target allows for larger horizontal motion and requires frequent shifts in light direction to keep the tip in position, as observed in the real plants in **(A)**.

We showcase the model mimicking the behaviour of the closed-loop bean tip controllers artificially evolved in Hofstadler et al. (2017; Figure 11). The task is to guide a single growing and nutating tip through specific targets on the 2D plane of the camera projection during its (growth-)journey through the image. The two light sources in the system are both binary (either on or off) and mutually exclusive (one and only one is on at any given time). The plant tip is detected continually by image processing, and its position—along with the current target position—is passed to an artificial neural network that decides which side to light up. The light-emitting behaviour of the CASU control software that was retrieved by artificial evolution is simple: if the plant tip below is detected left of the current target, then turn on the right light and vice versa. Here, we directly implement this rule in the definition of the light ratio Λ(*t*).

To scale the model to the dimensions of the experiment, we first interpret the time axis as an approximation of the vertical position of the bean tip (assuming a constant growth rate and ignoring geometrical constraints caused by bean stems curved in 3D space). Second, we treat the inhomogeneity metric of flexible plant mass Υ(*t*), as defined in Eq. P-7, as the current horizontal position of the tip.

The target’s horizontal position Γ(*t*) is defined in the scale of the plant inhomogeneity metric Υ(*t*) ∈ (0,1) and then mapped to the time axis (in minutes) such that Γ(*t*) = 0.85 while 0≤*t*≤640, Γ(*t*) = 0.2 while 641≤*t*≤880, and Γ(*t*) = 0.5 while 881≤*t*≤1,200. To mimic the behaviour of the artificially evolved tip-guiding controller, we define the light ratio function Λ(*t*) as

(P-10) $$\Lambda \left(t\right)=\left\{ \begin{array}{c} 1\\ 0 \end{array}\right.\begin{array}{c} \cdots \text{if}\Upsilon \left(t\right)<\Gamma \left(t\right)\\ \cdots \text{else} \end{array}.$$

If the plant tip (Υ(*t*)) is left of the target’s horizontal position Γ(*t*), switch on the right light and vice versa. We do not include a term for the detection error *X*^detect^(*t*), because in the experiments modelled here, the tip detection via image processing worked almost perfectly.

The simulation starts with all system variables empty [i.e., $P^{\text{stem}}\left(0\right)=P_{L}^{\text{flex}}\left(0\right)=P_{R}^{\text{flex}}\left(0\right)=0.0$] and runs until time step *t* = 1,200.

An exemplary run of the simulation (with the parameters given in Figure 2C) is shown in Figure 11 next to the recorded history of a bean plant controlled by a neural network artificially evolved in Hofstadler et al. (2017). The model successfully produces trajectories closely resembling those of real plants in the showcased scenario, with larger variations in horizontal tip position, when the target is located centrally.

### The Next Step: Leaving the Lab and Bringing the Robots Into the Wild

To achieve our goal of stabilising ecosystems, the robots will have to leave the controlled laboratory conditions and interact with ecological keystone species in natural environments. The stimuli that were tested under laboratory conditions can serve as a starting point to allow the robots to interact with the animals. However, we assume that these stimulus patterns will then need to be further optimised to work in this out-of-the-lab context. Here, we show that influencing the decision making of an entire colony of honeybees is also possible outside of laboratory conditions. We take advantage of the dual nature of managed honeybee colonies: on the one hand, the western honeybee is a farm animal, bred for economic purposes, and cannot be considered a completely wild animal. Thus, many aspects of the colonies’ lives are already highly controlled by humans (e.g., hive location, hive volume and materials of the beehive); on the other hand, the animals live very self-sufficiently compared with other farm animals and organise and control themselves to a large extent autonomously (e.g., foraging location, foraging plant, and internal hive organisation). Therefore, we work with animals outside of laboratory conditions that have access to a natural habitat and interact with wild plants and animals, but still under relatively controlled conditions. The experiments described in this section show how subtle physical cues generated by technical means can alter the hive-internal behaviour, while maintaining the free access of the colony to a natural environment and foraging in the wild. Influencing certain hive-internal behaviours can directly modulate the colony’s interaction with the ecosystem. For example, foraging side information transfer by dance communication can be inhibited by introducing artificial dance recordings, reducing the recruitment of new foraging bees (Kirchner, 1993), or honeybee flight activity can be suppressed all together by introducing artificial substrate vibrations (Spangler, 1969).

**FIGURE 12:**
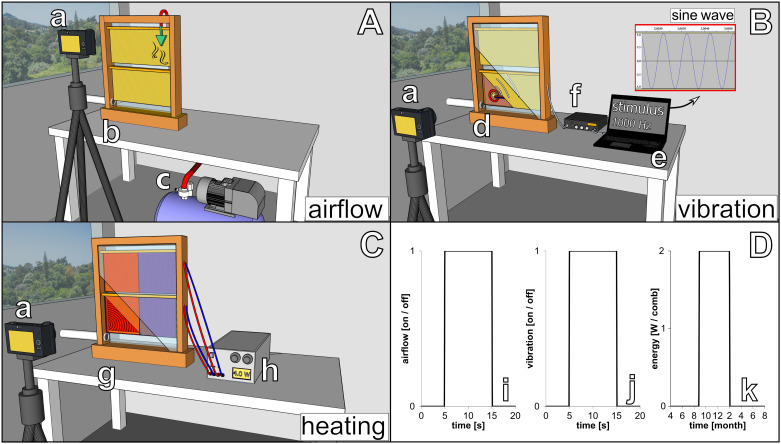
Set-up diagrams of three stimulus types used to influence the decision making of honeybees in a full colony. **(A)** Set-up for guided dispersal through airflow: (a) camera, (b) observation hive with airflow inlet, and (c) compressor. **(B)** Set-up for activity modulation by vibration signals: (a) camera, (d) observation hive equipped with piezo transducer, (e) stimulus generator, and (f) amplifier. **(C)** Set-up for influencing clustering behaviour through temperature signals: (a) camera, (g) observation hive equipped with heating elements, and (h) laboratory power supply. **(D)** Idealised stimulus time plot of (i) airflow, (j) vibration, and (k) energy input; actuation duration for airflow and vibration was 10 s, for heating 6 months.

**FIGURE 13:**
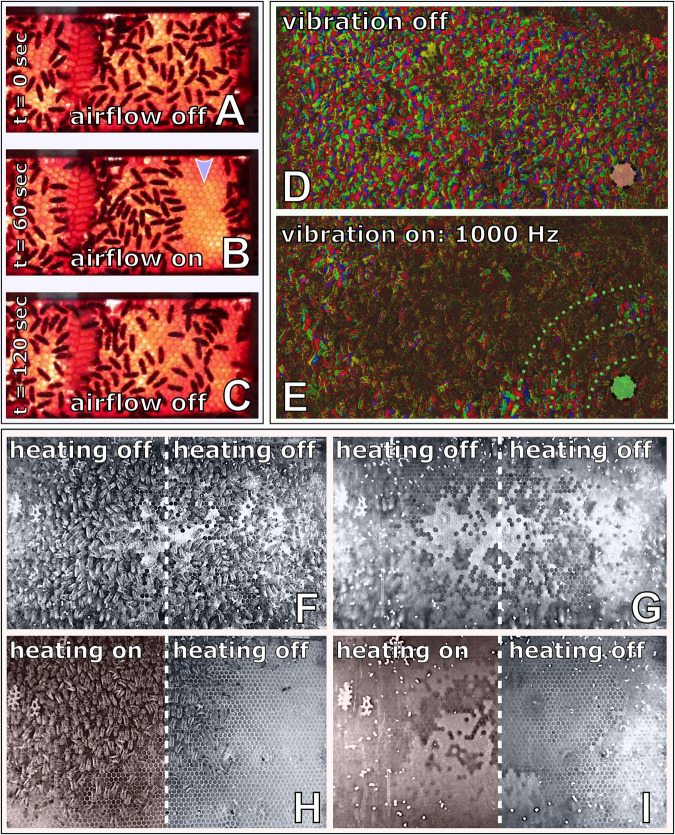
Effects of three stimulus types, which were first investigated on honeybees under laboratory conditions, now employed in the context of a full beehive in the wild. Subfigures show the effect of these stimuli in a “before/after” type comparison. (**A–C)** The guided dispersal through airflow: **(A)** the distribution of bees before the stimulus, **(B)** how the bees react to the stimulus (the arrow shows the location of the airflow), and **(C)** the bee redistribution after the stimulus has ended. **(D,E)** The activity modulation by vibration signals, visualising the movement on the honeycomb over three points in time (with a difference of approximately 2 s). Each colour channel (red, green, and blue) represents the bee positions at one point in time. A lot of movement results in a colourful picture; few movement results in a dark picture. **(D)** Normal movement on the honeycomb over a timespan of 4 s, no artificial vibrational signal. **(E)** A 1,000-Hz vibration signal that leads to significantly less movement over 4 s. **(F,G)** Influencing behaviour through temperature signals: **(F)** shows the bee distribution on a comb without active heat supply (day 0), bees are distributed over the entire honeycomb, **(G)** the distribution of the brood nest area, bright spots indicate capped brood cells containing larvae, distributed over the entire honeycomb (day 150). **(H)** The bee distribution on a comb with active heat supply on the left side (marked red, day 150); bees are mainly on the left honeycomb side. **(I)** The distribution of the brood nest area after active heat supply on the left side; bright spots indicate capped brood cells, predominantly on the left side of the comb (day 60). **(G,I)** Cells were made visible by background extraction of a stack of comb photos.

These experiments pose new challenges: the autonomous technical artefacts not only have to deliver precise stimuli to the animals, but they must also evaluate the behaviour of the animals under difficult conditions and, moreover, must be integrated into the environment in such a way that the regular organismic processes are not disturbed. For actively intervening in a honeybee colony, a more integrated form of “robot” is required. These robots have to be so pervasive in the colony that the whole honeycomb becomes a bio-hybrid robot. In order to achieve such a biohybrid system, we placed sensors and actuators in-between the areas accessible for bees (the comb surface). The airflow (900–950 cm^3^/s) is generated outside the hive and is introduced into the colony through a pipe (diameter = 4 mm); the used vibration stimulus patterns (sine wave, frequency = 1,000 Hz) are generated by thin piezo elements embedded in the wax comb and temperature stimuli (energy input = 2 W/comb, power density = 0.0053 W/cm^2^) are achieved by flat thermal elements in combination with small temperature sensors also embedded in the comb. More detailed diagrams of the experimental set-ups and additional information are given in Figures 12A–D. Figures 13A–I show the observed effects of these three stimulus types on an augmented honeycomb in a full honeybee colony. The airflow stimulus is shown to temporarily displace bees from certain locations on the honeycomb; the vibration stimulus is shown to influence the honeybees’ movement activity; and artificial energy input at certain positions of the comb is shown to influence the brood nest position. This system could allow to interrupt the dancing behaviour (by airflow or vibration stimuli) and thus alter the transfer of various sources of environmental information from outside the hive to the colony. Inhibiting certain behaviours could also lead to the increase of forager recruitment, in turn increasing pollination flights. The queen can also be prevented from laying eggs in the short term or at a specific location (either by airflow or vibration stimuli), or egg laying can be influenced in the long term by influencing in-hive temperatures. This in turn can modulate the growth of the bee colony.

These experiments show that, as a first step towards ecosystem stabilisation, in a full honeybee colony and outside of laboratory conditions, artificial stimuli can be used to influence certain behaviours of individual bees (through airflow or vibration stimuli) and of the colony as a whole (through artificial energy input). These honeybee behaviours that are responsive to robotic influence are related to the honeybee interactions with their ecosystem.

## Discussion

Human well-being crucially depends on strong, healthy and diverse ecosystems. The services that ecosystems offer us range from providing food from primary producers and from higher trophic layers to protecting our soils and cleaning our waters. They provide us with pharmaceuticals, energy, waste decomposition, climate regulation, and pest and disease control. And, last but not the least, they give us joy and inspiration, which we get from experiencing them all around us, inspiring us to arts and even science itself. For a sophisticated overview of dependencies between human society and ecosystem services, see Corvalan et al. (2005).

In this article, we described the severe problem of today’s ecosystem decay, and we identified central processes that are coupled in a vicious-cycle-type feedback loop that likely makes this problem auto-catalysing (Figure 1) as our key motivation to develop the hypothesis that autonomous robots could play an active role in slowing down or even reversing this decay in the future. In order to act in such a role, these robots will need to interact with living organisms in a way that allows them to influence the behaviour of groups or even populations of their living counterparts in a desired way. Thus, in some sense, these robots need to exert control over their organismic counterparts. We identified that social interaction might be one of the key factors here, as social systems tend to be self-organising systems where modest modulation of a few actors (Halloy et al., 2007; Bonnet et al., 2018) or of some small-scale local environment (Bonnet et al., 2019) can already change the collective local densities, which is known to be a fundamental factor in ecological interactions: It is a long-established fact that systems like predator–prey systems (Lotka, 1925; Volterra, 1926), host–parasite systems (Anderson and May, 1978), epidemic spread dynamics (Kermack and McKendrick, 1927), intra-specific competition (Verhulst, 1845), and inter-specific competition (Smale, 1976) are strongly driven by local population densities, not only affecting population dynamics but also relevant for their future configuration through natural selection (Hardin, 1960). In short, there is no ecologically relevant interaction amongst organisms that is not affected by the local density distributions of organisms. Recently, the field of robot–animal interaction studies has bloomed, also highlighting that robots are capable of affecting especially this factor, either by modulating aggregations or dispersal, or by directly influencing an organism’s motion behaviour.

Importantly, this characterisation highlights interesting pivotal points for novel types of intervention. We outlined how technological systems (autonomous robots and CASU arrays) interacting with biological collectives (swarms, societies, and communities) are able to influence specific natural processes (coordination, aggregation, growth, and activity levels), which ultimately affect ecosystem dynamics and stability. Thus, these technological artefacts may act upon the causal loop of ecosystem stability or decay. We outlined general approaches for bio-hybrid systems’ design, as well as the state of the art in the relevant scientific and technological progress. While we have not shown robots that actually repair ecosystems in the field in this study, we have been investigating the main prerequisites here to support our key hypothesis of possible robotic ecosystem stabilisation.

We demonstrated that robotic agents can modulate key organismic behaviours in a way that our family of models can predict concerning the collective dynamics across several empirical studies involving diverse species. Importantly, all three models share the same core structure to describe changes in decision making, comprising individual, and social processes. This commonality amongst the models indicates the feasibility of a more general application of such an organismic augmentation of natural societies with robotic agents in as-yet unexamined species, provided that analogous social dynamics and generatable signals can be identified. Additionally, the preliminary work towards modulating ‘‘wilder’’^3^ systems lends support to the technical feasibility of short- or long-term animal–robot interaction outside of laboratory environments, which could also be used as a bridge to exchange information between various ecosystems (Bonnet et al., 2019). Together, these prerequisites begin to form the foundations of a technology to allow us to test our key hypothesis: autonomous robotic agents can take a vital role in the preservation and stabilisation and maybe even in the repair of our precious ecosystems.

The first logical step towards rescuing ecosystems is not, of course, to just throw some robots at the problem. Instead, as many studies suggest, the first contingency policy must be altering human behaviour and collecting insights into the relevant ecosystems, and also into the relevant socio-economic systems that affect these ecosystems (Corvalan et al., 2005). For both, mathematical modelling, simulation and complexity science are important fields to understand these systems. Using automatic robotic probes for environmental monitoring (Schofield et al., 2010; Whitehead et al., 2014; Thenius et al., 2018) and population estimation (Le Maho et al., 2014; Vas et al., 2015) can be the first line of a robotics-based defence.

Robotic technologies have already been applied in ecological concerns, ranging from application of commercial drones (e.g., Vas et al., 2015) to special-purpose robot swarms (e.g., Thenius et al., 2018). In the latter, a swarm of (100+) autonomous robots was developed as a novel tool to observe large lagoon areas, even urban ones like the Venice lagoon. In this system, each robot is capable of reacting to its past measurements and potentially repositioning the swarm towards more interesting locations. These robots interact with microbial life forms in order to generate the required energy and, thus, are self-sustained for long operational times in an environmentally friendly way (Donati et al., 2017; Thenius et al., 2018). Using mud as an energy source enabled autonomous operation for several months (Kumar et al., 2018), a very interesting and eco-friendly power supply method for robots in the context we discuss here.

However, just monitoring and analysing might not be enough. At some point, intervention might be a necessary step in the contingency. There are alternatives to using autonomous robots; however, the ones most often discussed are not unproblematic: Genetic alteration of existing species is one contingency often discussed but also often criticised due to the dangers that come with it (Marvier, 2001; Devlin et al., 2015). Sometimes, ecosystem restructuring is discussed (and partially already done) by bringing specific species from other habitats in order to achieve desired effects, for example, in “biological pest control” (Hajek and Eilenberg, 2018). However, as we have learned from a rich history of problems that occurred with invasive species, also this contingency strategy is a dangerous path to go (Simberloff and Stiling, 1996; Henneman and Memmott, 2001). One imminent threat is that in both of these cases the “ecological agents” are capable of reproducing and adapting, and thus they are capable of spreading in an uncontrolled manner and, in parallel, of altering their original properties in the novel environment over time. This is a risk that does not exist in robotics, as the production of these devices can be centralised in contrast to decentralised self-reproduction of organisms, and updates can be deployed rapidly in the field via GSM or other technology, eliminating mal-adaptations as soon as they are detected. However, it will require solving other problems: The first relates to long-term robotics in the field (Yang et al., 2018), such as material recycling, self-repair (Kriegman et al., 2019), and self-healing (Terryn et al., 2017), which aim to maintain functionality even if failures occur or reduce the risks of failure while deployed, sources and storage of energy (Kumar et al., 2018), and in principle a more environmentally friendly and sustainable set of materials and technology. In this last respect, advances in manufacturing and materials sciences such as the use of organic substrates in semiconductors (Torsi et al., 2013) and computing elements (van de Burgt et al., 2018), and recent techniques combining 3D printing of ceramics and moulding of more biocompatible materials (Puppi and Chiellini, 2020) are all promising directions. The second relates to biocompatibility, which is essential for the robotic agents to successfully intervene in an ecosystem (Baumgartner et al., 2020). Third, focussing on one keystone species, as we have argued, is the natural place to start, but more complex networks of biology and technology are likely necessary.

Even though a robotic ecological agent does not suffer from the same issues as the biological interventions discussed above, the use of technology in ecology raises several ethical concerns. It is thus essential to be clear about the methods to be used. Measuring stress levels and welfare in animals is a non-trivial task (Dawkins, 2003), and although it is certainly on the mind of some designers of bio-interacting robots (e.g., Le Maho et al., 2014; Vas et al., 2015), systemic ethical treatments are rare, as they are still in their infancy (Donhauser et al., 2020). We have argued above for robots to only emit stimulus types and intensities that occur in the organism’s natural environment and that have no known negative side effects on the organisms. This limitation is based not only on ethical considerations but also on ecological ones. Using stimuli that are outside this natural range would potentially be incompatible with the perception and response capabilities of the individual and could potentially bring the society into a state that is unknown and not coherent with its ecosystem, which is exactly what we try to avoid.

As soon as the plan is to leave the controlled environment, e.g., the lab, and to take the robots out into the wild, more ethical considerations must be made. There are questions regarding who is responsible in the case of a system failure (Grémillet et al., 2012) or for maintaining technology that supports an ecosystem (Donhauser et al., 2020). Moreover, the potential disturbance caused by robotic devices during their operation (Le Maho et al., 2014) and after a system failure (Borrelle and Fletcher, 2017) are important concerns, which may be partially addressed through biocompatible design and biodegradable material choices, as noted above. There are some valuable lessons from the retrieval of bio-sensors after deployment (see e.g., Fossette et al., 2016). More generally, self-monitoring and identification of system degradation could be used to trigger a retrieval of the robot before failures result in unrecoverable devices polluting the environment intended to be supported. Although a robot’s ability to integrate into biological societies is usually emphasised (e.g., Papaspyros et al., 2019), a mode in which the reverse is emphasised, i.e., a non-influencing mode, could be employed to depart an animal collective with minimal disruption. Even more fundamental questions have to be asked and answered in future research: Do we understand enough about the effects that populations, modulated by robots, will have in the environment? Can we observe what is going on, in order to monitor the efficiency of the new biohybrid system and to detect potential side effects? Can the system be restored to full self-sufficiency, and if so, what is the exit strategy? Else, how can we avoid the development—and possibly evolution—of a deepening dependency of the natural system on the robots? Is there a sufficient benefit to justify robotic intervention in the ecosystem, compared with the risks mentioned above that this intervention could induce on the ecosystem? For answering these questions, a profound knowledge of the modulated species and their ecological interaction partners is crucial, demanding sophisticated basic research on the physiology and ecology of these species and their interaction partners.

Social interaction offers an easy entry point for robots that they can exploit to engage with natural organisms. By modulating these social interactions, ecological key variables can very easily be affected, most prominently population densities, which in turn affect competition rates, mate-finding rates, and also the spreadi of parasites or infectious diseases. Each of these issues has received attention, but much is left to be done. Thus, modelling the modulation of social interactions by autonomous robotic systems is a key aspect to understand and predict such biohybrid interaction systems.

All three models that we have developed for predicting the dynamics emerging in the investigated biohybrid systems of robots associated with bees, fish, and plants have significant similarities amongst them, suggesting a sort of “common core” mechanism across this very diverse spectrum of organisms. Abstract ODE models of such systems have been used only rarely in the past, e.g., for describing a bio-hybrid set-up of cockroaches and robots (Halloy et al., 2007); however, our models presented here are significantly simpler given their level of non-linearity and the number of parameters to describe the animals’ behaviours, mainly describing a sort of homeostasis-like regulated system of diffusion of organisms. Despite some organism-specific differences, the striking similarity between all three models suggests that we have encapsulated a core principle of organismic population density control that can be used to allow robots to manipulate local organism densities.

*Simplicity and Wide Application:* Besides being all systems of ODEs that are numerically solved (see Figure 2E) that describe collective binary decision making (bees left vs. bees right, fish CW vs. fish CCW, and plants left vs. plants right; see Figures 2A–C,F), our three models all ensure conservation of mass within the reference frame they describe. The bee model and the fish model are both totally closed systems; and the plant model has one defined entry (source) and two defined exit points (sinks), and full mass conservation between these processes. When applied to larger populations on the long term, there will surely be a need to extend these models to allow additional biomass influx (reproduction) and outflux (death) with respect to the modelled systems. The basic model structure (Figure 2F) allows for separating specific ecologically relevant behavioural processes within the natural organism populations. For example, by adjusting the ratio of α:β, the specific contribution of individual (α) and social behaviour (β) can be adjusted in the systems in all modelled species. These parameters govern the weight of terms that are modelling natural processes that are affected by noise and the relevant stimuli (see Figures 2D,F). In each of the social interaction equations of the different organism groups (Eqs B-3, F-6, P-5), there are two constant parameters that define the ratio of exploitative (β) and explorative behavioural components (σ). Adjusting the ratios of β:σ allows the model to capture the exploitation–exploration trade-off of specific organism groups or species. In consequence, by varying the ratio of all three parameters together, α:β:σ, the model can predict the ultimate macroscopic effects of a rich set of microscopic behavioural repertoires in a rather simple system of ODEs, including the modelling of the effect of robotic actors within the system. These striking similarities between all three models suggest that we have encapsulated a core principle of organismic population density control that can be used to allow robots to manipulate local organism densities. The simplicity of the modelling approach is also valuable because it can guide what factors robots should modulate and in which direction. For example, a mechanism for guided aggregation will adjust the social switching parameter, while guided locomotion could affect the β:σ ratio.

*Downsides of Simplicity:* The simple approach to modelling naturally yields some limitations in how much of the dynamics can be captured. As is the case with most ODE models, no population structure is modelled; i.e., it is considered as freely mixed, for example, concerning age, sex, health, and other physiological states. The broad trends are well captured, but the variability that typifies organismic behaviour is not present in the model results presented above. We consider this to be one of the main reasons why our model predicts a significant lower variance in local population dynamics than observed in the empirical experiments. Typical for ODE models, agents are modelled as infinitesimally small; thus, effects like traffic jams cannot occur if not explicitly modelled into the equations. Also typical for ODE models, interaction and sensing of the modelled entities are not limited *per se* to a limited range, again allowing more coherent action and thus lower variations.

Elsewhere, we have employed individual-based modelling for some of these bio-hybrid systems that shows more variability (e.g., Mills et al., 2015; Stefanec et al., 2017a), but at the cost of generality.

The lack of observable variance predicted in converged situations of the described systems can also be due to the simplicity of our model approach. On the one hand, the model might exhibit a larger variance if it contained a third stock variable representing the undecided, thus more diffusing organisms, like it was modelled in Schmickl et al. (2009a, b) and Kernbach et al. (2009). On the other hand, even such an extended model can still exhibit a low variance in its predictions, due to the implicit base assumptions of ODE models in principle, such as the assumption of optimal mixing and distribution of the modelled agents in space within the areas modelled by each system variable. In this case, a step to spatially explicit individual-based models and spatially more heterogeneous models, like cellular automata (Szopek et al., 2017) or multi-agent models (Stefanec et al., 2017b), might be more suitable to capture the effects of higher variances that are often observed in natural, and thus physically manifested, systems.

*Actionability:* We went beyond the usual benefits of mere modelling and beyond the three specific biohybrid systems that we touched in this article. In our methodological approach, mathematical models of biohybrid systems serve a significantly deeper purpose: the predictions and analyses of such mathematical models allowed us to identify which natural reactions of the organism are the best to be utilised as “social interaction hooks,” most likely allowing the robots to blend into the natural organismic system. Thus, these models suggest promising robot design directives by indicating how the principles of guided aggregation and guided locomotion can be implemented as a set of microscopic mechanisms of the robots in order to exert the desired control of specific macroscopic key variables of the collective system, e.g., local density or group motility. These variables are known to have significant effects on many important ecological processes, such as competition, reproduction, parasitism, and mutual reciprocity (symbiosis). We found that the type of mathematical models that we present here, which are rather simple and thus abstract, already proves quite helpful, as they sufficiently predict the macroscopic group-level dynamics emerging from individual microscopic actions that are executed in parallel and in a distributed manner. Thus, even such simple models already inform us which variables to adjust in the individual robots’ behaviours in order to exploit the appropriate set of cues in the system to ultimately achieve the desired group-level dynamics and system properties.

**FIGURE 14:**
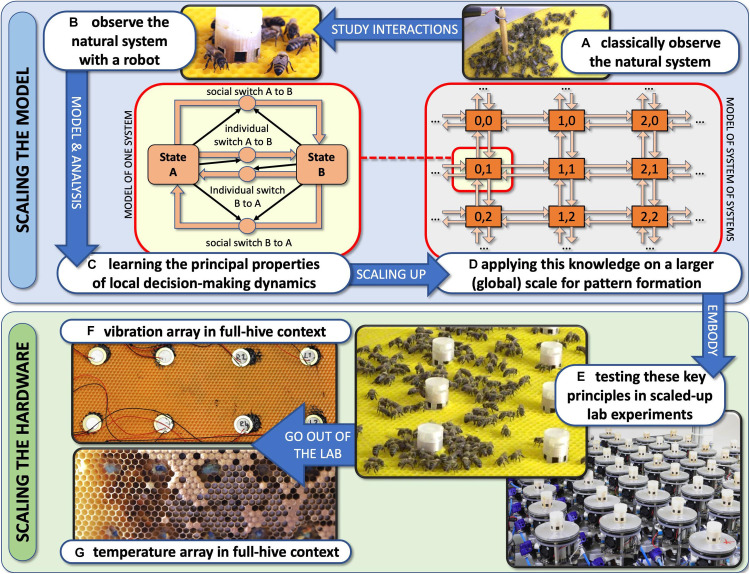
Summary of the work process that we suggest for developing ecologically relevant autonomous robotics. **(A)** Observing the interaction patterns of organisms. **(B)** Studying their reactions to stimuli emitted by robots and also the robot’s sensing capabilities for relevant environmental configurations. **(C)** Describing these interactions in small-scale specific models to identify relevant core principles that can be used for larger-scale pattern formation. **(D)** Scaling these models up to larger, thus more relevant, sizes. **(E)** Testing scaled-up pattern formation in specific hardware equipment under laboratory conditions in order to test the validity of the scaled models. Finally applying the behavioural modulation on the targeted size- and time-range (in our case a full honeybee comb over weeks or months) to employ specific stimulus patterns to be used to interact with the target organism population, e.g., comb vibration **(F)** or temperature distributions **(G)**.

*Scalability:* In our article, we have first described small-scale experiments that were conducted in the form of binary decisions. This is the smallest relevant system, as its state space can be compressed into 1 bit of information in order to sufficiently describe it. These small-scale experimental models allowed us to generate small-scale mathematical models that were sufficiently accurate in predicting the systems’ final state and the time dynamics of state changes. These building blocks can then be used to find out which physical properties have relevant effects that will potentially also operate on the larger scale. This scaling-up prediction can be derived from using our simple systems of ODEs to construct larger systems of ODEs. Such a model would take a “system of systems” perspective of a larger space. For example, the model could arrange the ODE-based building blocks into a lattice where each node in the lattice is one small-scale ODE system that is interacting with its local neighbour systems via diffusion flows. These flows can represent the motion (taxis or tropisms) of the modelled organisms. After appropriate robotic regimes for the desired pattern formation induced within the organismic population were found, these principles can be tested under laboratory conditions by larger robot swarms or arrays to see if they also work as expected in a larger-scale physical implementation. Finally, such systems can be applied with organisms that interact with other organisms “in the wild,” as we demonstrated with honeybees as a proof of principle in section “The Next Step: Leaving the Lab and Bringing the Robots Into the Wild.” Figure 14 gives an overview of a 10+ years’ research track that we started with simple honeybee experiments with young baby bees in laboratory conditions with two fixed heat lamp spots or with two simple vibration motors taken from cell phones (Figure 14A, diverse other set-ups not shown here; see for example, Scheiner et al., 2013), via a robot that can emit such stimuli autonomously and can exhibit its own agency (Figure 14B), to a model of two such robots (Figure 14C), to a scaled-up model depicting the dynamics across larger areas (Figure 14D), to a full array of 64 autonomously acting robots (Figure 14E), to finally be implemented on combs of a full-fledged honeybee colony that successfully forages for pollen and nectar in the environment being affected via a comb-embedded system of such stimuli—emitters and sensors (Figures 14F,G).

Having such autonomous robots weaving additional and controllable interaction threads into the fabric of natural ecosystems might, in the future, allow the stabilisation of endangered ecosystems that lost their intrinsic resilience due to anthropogenic influences like global warming, industrial pollution, over-harvesting, or massive farming. To get such biohybrid systems operational and exhibiting the desired ecological effect without a human in the loop curating the system will be an extremely challenging task. It will require important progress in robotic biocompatibility, autonomy, flexibility, and energetic efficiency, as well as towards robotic robustness and resilience. In contrast to almost all technical artefacts that we know of today, natural organisms can heal, reproduce, and adapt. All these features help them to survive in the wild and are thus crucial for spreading and covering large habitats. The state of the art in autonomous robotics in these domains is far from a level of sophistication that would allow us to spread robots without human intervention and curation on a comparable long-term and large scale. Ultimately, the creation of such ecosystem-stabilising robotic systems is a far-reaching goal, which we all hope not to be needed in the end, as we hopefully manage to stabilise and repair our earth’s ecosystems with more conventional methods. However, if we will need such a technology to save or support our ecosystems, the relevant research is just in its early stages, and producing effective robots might take decades of research. To operate such systems safely for humans and for nature, we think that much research on organisms, robots, and algorithms is still required. In our opinion, research in these topics must expand now, in the context of allowing robots to operate in natural habitats, for us to be ready to employ them in case we might need them in the future.

## Data Availability Statement

The raw data supporting the conclusions of this article will be made available by the authors, without undue reservation.

## Ethics Statement

For the experiments with invertebrates including honeybees conducted in Austria, no restrictions to experimentation apply, and no specific ethical board approval of experiments is required. For the experiments with zebrafish, the study was reviewed and approved by the state ethical board for animal experiments under authorisation number 2778 from the DCVA of Canton de Vaud, Switzerland.

## Author Contributions

TS developed the core hypothesis developed in this manuscript, conceived the basic line of research outlined here, and implemented the first models on bees, fish and plants, and robots. The models were then strongly further elaborated mainly by PZ (especially the fish model), but the honeybee model was also scientifically improved by RM, MSt and DL together with TS. The plant model was further improved by DH together with TS. TS, MSz, RM, FB, FM, MSt, DL, RB, DH, and PZ wrote the text of the manuscript together in a collaborative effort. Experiments B1 and B4 were conducted by MSt and MSz (honeybee experimentation) and RM (CASU control and data analysis). Experiment F1 was conducted and analysed by FB and FM. Experiments F2 and F3 were conducted and analysed by FB, RM, and MSz. MSt, RB, and RM designed the materials and conducted experiments shown in Figures 12, 13. MSz performed the data collection and analysis of the empirical data of B2. MSz and MSt performed the data collection and analysis of the empirical data of experiments B1 and B4. Figures 1, 2, 4, 14 were conceived and implemented by TS. Figure 3 was conceived by FM. Figures 5 and 6 were conceived and implemented by MSz. Figures 7, 8, 10 were conceived and implemented by MSt, DL, RM, and PZ, with input from RB, FB, and TS. Figure 9 was conceived and implemented by FB. Figure 11 was conceived and implemented by DH (with input from TS). Figures 12, 13 were conceived and implemented by MSt. All authors contributed to the article and approved the submitted version.

## Conflict of Interest

The authors declare that the research was conducted in the absence of any commercial or financial relationships that could be construed as a potential conflict of interest.

## Footnotes

1 In a mean-field model, like this ODE model, the model expresses the mean time budgets of fish swimming in either direction, so fractional quantities are not unrealistic.

2 We used the forward Euler integration method instead of the Runge–Kutta method to solve Eqs P-1a–c; thus, for the plant model, we use difference equation notation, instead of the differential equation notation that was used for the bee and fish model. Runge–Kutta integration was precluded by the non-differentiable binary switching of the lights.

3 We discriminate between “in the lab” experiments, which we analysed and modelled here, and “in the wild” applications, which we target in our current research tracks, based on the results that the previously conducted laboratory experiments yielded.
